# Comparative Transcriptome Analysis of CMV or 2b-Deficient CMV-Infected *dcl2dcl4* Reveals the Effects of Viral Infection on Symptom Induction in *Arabidopsis thaliana*

**DOI:** 10.3390/v14071582

**Published:** 2022-07-21

**Authors:** Qian Xu, Li Shen, Liying Jin, Meng Wang, Fenghan Chang, Zhongxin Guo

**Affiliations:** Vector-Borne Virus Research Center, State Key Laboratory for Ecological Pest Control of Fujian and Taiwan Crops, College of Plant Protection, Fujian Agriculture and Forestry University, Fuzhou 350002, China; 254491112sl@sina.com (L.S.); jinliying2021@163.com (L.J.); iswang_meng@163.com (M.W.); 17750259325@163.com (F.C.)

**Keywords:** *Arabidopsis thaliana*, cucumber mosaic virus (CMV), *dcl2dcl4* (*dcl2/4*), transcriptome

## Abstract

Due to the impaired antiviral RNAi, the *dcl2dcl4* (*dcl2/4*) mutant is highly susceptible to viruses deficient of the viral suppressor of the RNA silencing (VSR) contrast to wild-type Arabidopsis. It was found that more severe disease symptoms were induced in *dcl2/4* infected with VSR-deficient CMV (CMV-Δ2b or CMV-2aTΔ2b) compared to wild-type Arabidopsis infected with intact CMV. In order to investigate the underlying mechanism, comparative transcriptome analysis was performed with Col-0 and *dcl2/4* that were infected by CMV, CMV-Δ2b and CMV-2aTΔ2b, respectively. Our analysis showed that the systematic infection of CMV, CMV-Δ2b and CMV-2aTΔ2b could cause hypoxia response and reduce photosynthesis. Asymptomatic infections of CMV-Δ2b or CMV-2aTΔ2b in Columbia (Col-0) promoted the expression of cell division-related genes and suppressed the transcription of metabolism and acquired resistance genes. On the other hand, immunity and resistance genes were highly induced, but photosynthesis and polysaccharide metabolism-related genes were suppressed in diseased plants. More interestingly, cell wall reorganization was specifically caused in modestly diseased Col-0 infected by CMV and a strong activation of SA signaling were correspondingly induced in severely diseased *dcl2/4* by CMV or CMV mutants. Thus, our research revealed the nature of the Arabidopsis–CMV interaction at the transcriptome level and could provide new clues in symptom development and antiviral defense in plants.

## 1. Introduction

Viruses are biotrophic parasites that must usurp host factors for their propagation. Pathogenesis usually occurs when plant virus overcomes plant defense to infect and causes detrimental effects to the host. Pathogenic viruses have a severely negative impact on agricultural production worldwide [1]. Establishing effective strategies for minimizing the damage of virus epidemics will require understanding the responses of plant hosts to viral infection. Given the broad host range and the detrimental effect on agriculture economy in worldwide, CMV is regarded as an important crop pathogen and also as a model virus to study plant–virus interactions [2]. CMV is commonly found in wild populations of Arabidopsis at up to 80% prevalence [3], and, therefore, the Arabidopsis–CMV interaction is relevant in nature.

*Arabidopsis thaliana* has been widely employed to investigate host responses to viruses because of its advantages for molecular genetic approaches. Through plenty of genetics analysis and function characterization on the infection of Arabidopsis by viruses, including but not limited to cucumber mosaic virus (CMV), turnip mosaic virus (TuMV), turnip yellow mosaic virus (TYMV), turnip crinkle virus (TCV) and cauliflower mosaic virus (CaMV), large amount of host factors with antiviral activity against plant viruses have been identified [4,5,6,7,8,9,10,11,12,13].

The RNAi-based antiviral defense is a fundamental antiviral innate immunity in plants to restrict virus replication and movement by slicing viral RNAs and inhibiting viral protein translation [14,15,16]. In the process, Dicer-like enzymes (DCLs) recognize long double-stranded viral RNAs as pathogen-associated molecules and process them into 21–24 nucleotide (nt) small interference RNAs (namely virus-derived siRNAs, vsiRNAs) [17,18,19]. Subsequently, antiviral Argonaute proteins (AGOs) bind vsiRNAs to mediate either translational repression of complementary target mRNAs or transcriptional silencing of target DNAs [20]. In addition, two of six Arabidopsis RNA-dependent RNA polymerases (RdRPs), RDR1 and RDR6, efficiently synthesize double-stranded RNAs to generate secondary vsiRNAs through DCLs [21]. Thus, vsiRNAs are directly produced through the processing of DCLs. Arabidopsis has four DCLs, which function redundantly or cooperatively in the antiviral immunity [17,18]. DCL4 is the major player responding to the RNA virus in Arabidopsis, and DCL2 can subrogate DCL4 when the infected host is with the compromised DCL4 function [18]. In this context, only plants with null mutation on both DCL2 and DCL4 are susceptible to infection with CMV or other plant viruses and develop severe disease symptoms [5,22].

To counteract host antiviral silencing, most viruses have evolved viral suppressors of RNA silencing (VSRs) independently. VSRs from different viruses target distinct steps of the antiviral defense, such as blocking vsiRNA biogenesis by binding to viral dsRNAs or interacting with host DCLs, DsRNA binding protein (DRB), RDRs or the suppressor of gene silencing 3 (SGS3), preventing AGO-vsiRNA/RISC formation or function by sequestrating duplex siRNAs or disturbing the functions of AGOs, and limiting the spread of the silencing signal by disrupting the movement of viruses and vsiRNAs [16,23]. As one of the earliest documented VSRs, CMV 2b could directly bind both double-stranded siRNA and AGOs to suppress RNA-directed DNA methylation and posttranscriptional gene silencing (PTGS) [24,25,26,27]. Besides, CMV 2b is a determinant of virulence and controls the severity of symptom in plants [28,29]. CMV 2b causes developmental anomalies in Arabidopsis, which phenocopies *ago1–25* and *ago1–27* [27]. CMV with 2b deletion results in the symptomless infection of wild-type tobacco and Arabidopsis but causes severe disease symptoms in mutants defected in vsiRNA biogenesis [10,30,31]. Thus, CMV-Δ2b or CMV-2aTΔ2b, two 2b deficient CMV, has been employed to screen critical components of the RNAi-dependent antiviral immunity [10,32,33]. These analogical symptoms of the antiviral RNAi defective mutants infected with CMV-Δ2b or CMV-2aTΔ2b imply that there should be commonly disturbed host factors that are affected by CMV independent of 2b and contributed to the disease symptoms. However, most studies focus on the elucidation of antiviral RNAi against viral infection pathways, and much less attention was paid to dissecting endogenous biological processes in host plants disturbed by CMV-Δ2b or CMV-2aTΔ2b infection.

In this work, we analyzed the transcriptome of Columbia (Col-0) and *dcl2dcl4* infected with CMV, CMV-Δ2b, or CMV-2aTΔ2b. Gene Ontology (GO) enrichment with differential expression of genes (DEGs) in different virus-infected plant showed that CMV, CMV-Δ2b and CMV-2aTΔ2b could generally induce hypoxia stress response and inhibit photosynthesis or chloroplast organization in Arabidopsis. Our further analysis demonstrated that asymptomatic infections of CMV-Δ2b or CMV-2aTΔ2b in wild type accompanied by the elevated expression of mitosis-related genes and the diminished transcription of chemical homeostasis and acquired resistance genes. However, symptomatic infections induced bacterium immunity and inhibited photosynthesis and carbohydrate metabolism. Interestingly, we found that mildly symptomatic CMV-infected Col-0 specifically displayed the induction expression of cell wall reorganization genes but severely symptomatic infected *dcl2/4* specifically displayed a strong activation of salicylic acid (SA) signaling. Thus, our findings shed light on the interaction among intact or VSR-deprived CMV and wild-type or antiviral RNAi-deficient plants for better understanding the underlying molecular mechanism.

## 2. Materials and Methods

### 2.1. Plant Growth and Virus Infection

*Arabidopsis thaliana* ecotype Columbia (Col-0) was used as wild type and mutant *dcl2dcl4* (*dcl2/4*) was described previously [33]. After synchronized at 4 °C in dark for 2 days, Arabidopsis were grown on soil at about 24 °C with 16 h light, 8 h dark cycle. The light intensity is about of 5000 lx. The virus used in this study is the Fny strain of CMV and the mutant virus CMV-Δ2b and CMV-2aTΔ2b, which are deprived of 2b, was described previously [33]. Purified virions propagated in *Nicotiana clevelandii* were used as the inocula at the concentration of 10 µg/mL. Plants about 25 days after germination were inoculated with inocula by the mechanic rubbing method.

### 2.2. Transcriptome Sequencing

Non-inoculated systemically infected leaves of mock plants without viral infection and Col-0 or *dcl2/4* infected with CMV, CMV-Δ2b or CMV-2aTΔ2b (simplified as Col-Mock, Col-CMV, Col-Δ2b, Col-2aTΔ2b, *dcl2/4*-Mock, *dcl2/4*-CMV, *dcl2/4*-Δ2b, *dcl2/4*-2aTΔ2b) were sent to BGI for transcriptome sequencing. Each sample was sequenced with three independent libraries. Library construction and transcriptome sequencing were accomplished by BGI. BGI performed the basic analysis and supplied clean reads.

### 2.3. Mapping and Quantification Differentially Expressed Genes (DEGs)

All the clean reads from Col-0 group and *dcl2/4* group were separately mapped back to the reference sequences (Arabidopsis_thaliana.TAIR10.50.gtf, https://www.arabidopsis.org/index.jsp (accessed on 19 April 2021) by SATR (https://github.com/alexdobin/STAR (accessed on 19 April 2021). Count reads were quantified by featureCounts (http://subread.sourceforge.net/featureCounts.html (accessed on 19 April 2021). FPKM (fragments per kilobase of exon million mapped sequence fragments) was used to calculate by “GenomicFeatures” R package (https://github.com/Bioconductor/GenomicFeatures (accessed on 30 April 2021) and the formula was as follows:

FPKM = Total Exon Fragments/[Mapped Reads (million) × Exon Length (kb)](1)

The FPKM was used to do PCA analysis by “FactoMineR” [34]. Genes that differentially expressed between the mock control plants and virus-inoculated plants were screened using DESeq2 [35]. The threshold of differentially expressed genes was set to: the up-regulated gene as log_2_FoldChang > 1 and Padjust-value < 0.05; the down-regulated gene as log_2_FoldChange < −1 and Padjust-value < 0.05.

### 2.4. Gene Ontology (GO) Enrichment Analysis and Venn Analysis

Gene Ontology (GO) annotations of the contigs were determined using ClusterProfile [36] according to the molecular function, biological process, and cellular component ontologies (http://www.geneontology.org/ (accessed on 22 April 2022). GO-enrichment analysis of DEGs was performed using the ClusterProfile R pock [36], and the Padjust-value were calculated using the Benjamini–Hochberg (BH) correction. Compared to mock plants, genes with Padjust-value < 0.05 were included in our analysis. The overlapping induced or suppressed genes were analyzed (https://xlinux.nist.gov/dads/HTML/venndiagram.html (accessed on 7 May 2022).

### 2.5. Gene Expression Analysis

The systemically infected leaves of 10 to 15 plants were pooled for RNA extraction two weeks after inoculation. The first-strand cDNA was synthesized from 1 ug total RNA with HiScript@ II Q RT SuperMix for qPCR (R233-01, Vazyme) according to the user manual. Real time quantitative PCR (qPCR) was performed on a CFX 96 real-time PCR detection system (BioRad, Hercules, CA, USA) according to the Taq Pro Universal SYBR qPCR Master Mix (Q712-02/03, Vazyme). The PCR reactions were subjected to an initial denaturation step of 95 °C for 30 min, followed by 39 cycles of 95 °C for 15 s and 60 °C for 30 s. The *actin2* gene was used as an internal control for studying the expression level of target genes. Relative gene expression levels were analyzed with 2-ΔΔCT method [37]. All reactions were performed in three technical and biological replicates. The primers for target genes were designed respectively by qPCR Primer Database (https://biodb.swu.edu.cn/qprimerdb/, accessed on 13 May 2022). The detailed information of primers showed in Table S1.

## 3. Results

### 3.1. Sequencing and De Novo Assembly of Transcriptome

**Figure 1:**
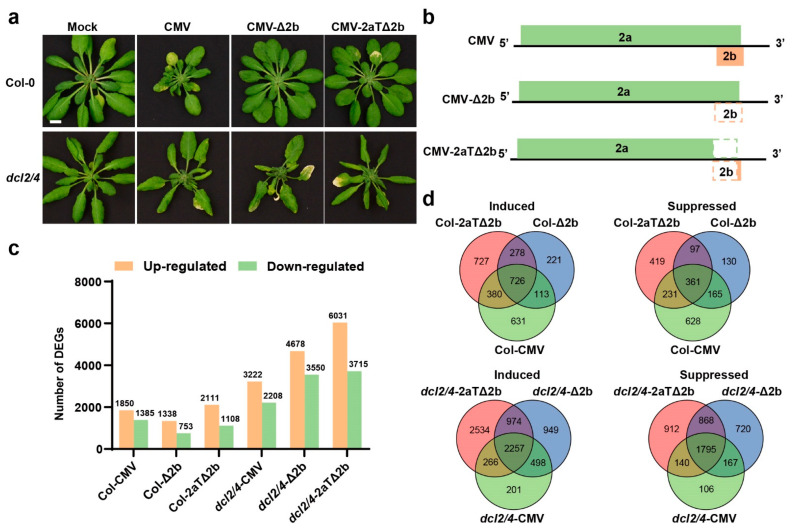
Development and transcriptome of Arabidopsis that were affected by cucumber mosaic virus (CMV) infection. (**a**) Phenotype of Col-0 and mutant *dcl2dcl4* (*dcl2/4*) inoculated with CMV, CMV-Δ2b and CMV-2aTΔ2b. Bar = 1 cm. (**b**) The RNA2 genome structures of CMV, CMV-Δ2b and CMV-2aTΔ2b. (**c**) Number of differentially expressed genes (DEGs) in CMV-, CMV-Δ2b-, and CMV-2aTΔ2b-infected plants (Col-CMV, Col-Δ2b, Col-2aTΔ2b, *dcl2/4*-CMV, *dcl2/4*-Δ2b, and *dcl2/4*-2aTΔ2b) compared to control plants (Col-Mock and *dcl2/4*-Mock). (**d**) Venn diagram analysis of genes number of differentially expressed genes (DEGs) among different gene sets in CMV-, CMV-Δ2b-, and CMV-2aTΔ2b-infected plants.

The deletion of VSR 2b leads to a reduction on virulence of cucumber mosaic virus (CMV). Systematically infected with 2b deficient CMV, Arabidopsis ecotype Columbia (Col-0) displayed no symptoms (Figure 1a) [38]. In contrast, double mutant *dcl2dcl4* (*dcl2/4*) is susceptible to CMV with or without its VSR, 2b protein (Figure 1a) [5], which is phenocopied by antiviral RNAi deficient mutants [10,21,31,32,33]. To obtain a comprehensive view of the transcriptome changes of Arabidopsis in response to CMV infection, the expression profiles of Col-0 or *dcl2/4* infected with CMV, CMV-Δ2b or CMV-2aTΔ2b (Figure 1b) (named as different groups Col-CMV, Col-Δ2b, Col-2aTΔ2b, *dcl2/4*-CMV, *dcl2/4*-Δ2b and *dcl2/4*-2aTΔ2b, respectively) were compared to mock control plants (Col-mock or *dcl2/4*-mock) by high-throughput RNA sequencing (RNA-seq). Low-quality reads and adapter sequences be removed, over ten million clean reads of RNA-seq were obtained in the Col-0 plant group (Col-Mock, Col-CMV, Col-Δ2b and Col-2aTΔ2b) and *dcl2/4* group (*dcl2/4*-Mock, *dcl2/4*-CMV, *dcl2/4*-Δ2b and *dcl2/4*-2aTΔ2b), respectively (Table S2). Principal component analysis (PCA) showed that all repeats of different groups shared a high co-relationship (Figure S1). After all the reads of the different groups were mapped to reference genome, gene mapping rate was between 91.99% and 96.84% (Table S2), indicating the high quality of transcriptome sequencing.

### 3.2. Comparison of the Differential Transcriptome between CMV-Infected and Mock Plants

To identify differentially expressed genes (DEGs) responding to virus infections, we firstly compared the virus-inoculated group to the mock group using DESeq2 [35]. Compared to Col-Mock plants, 1850, 1338, 2111 genes were induced and 1385, 753, 1108 were suppressed in Col-CMV, Col-Δ2b, and Col-2aTΔ2b, respectively (Figure 1c). Relatively, CMV-Δ2b triggered less DEGs than CMV and CMV-2aTΔ2b in wild type. Among these DEGs, 728 induced genes and 361 suppressed genes were overlapped in Col-0 group (Figure 1d). Gene Ontology (GO) analysis showed that host genes related to hypoxia stress response (GO:0001666, response to hypoxia; GO:0036293, response to decreased oxygen levels; GO:0070482, response to oxygen levels; GO:0071456, cellular response to hypoxia; GO:0036294, cellular response to decreased oxygen levels; GO:0071453, cellular response to oxygen levels), cell cycle process (GO:0022402), response to nitrogen compound (GO:1901698), anchored component of membrane (GO:0031225), cytoskeleton components and activity (GO:0005856, cytoskeleton; GO:0099080, supramolecular complex; GO:0015630, microtubule cytoskeleton; GO:0099081, supramolecular polymer; GO:0099512, supramolecular fiber; GO:0099513, polymeric cytoskeletal fiber; GO:0005874, microtubule; GO:0008092, cytoskeletal protein binding; GO:0008017, microtubule binding; GO:0015631, tubulin binding; GO:0003774, cytoskeletal motor activity; GO:0003777, microtubule motor activity) and protein kinase regulator activity (GO:0019887, protein kinase regulator activity; GO:0019207, kinase regulator activity) were induced by CMV, CMV-Δ2b, and CMV-2aTΔ2b (Figures S2–S4). Host genes associated with photosynthesis or chloroplast structure (GO:0015979, photosynthesis; GO:0006091, the generation of precursor metabolites and energy; GO:0009523, photosystem II; GO:0031976, plastid thylakoid; GO:0009534, chloroplast thylakoid; GO:0034357, photosynthetic membrane; GO:0042651, thylakoid membrane; GO:0055035, plastid thylakoid membrane; GO:0009535, chloroplast thylakoid membrane; GO:0010287, plastoglobule; GO:0009521, photosystem; GO:0031977, thylakoid lumen) and abiotic stress (GO:0009409, response to cold; GO:0006979, response to oxidative stress), and apoplast (GO:0048046) were suppressed by CMV and 2b-defecient CMV (Figures S2–S4).

In consistent with the severe disease symptom in *dcl2/4*, a much larger amount of genes were affected in *dcl2/4* upon viral infection. Compared to *dcl2/4*-Mock, there were 3222, 4678, 6031 genes induced and 2208, 3550, 3715 suppressed in *dcl2/4*-CMV, *dcl2/4*-Δ2b, *dcl2/4*-2aTΔ2b, respectively (Figure 1c). Meanwhile, 2257 induced genes and 1795 suppressed genes were overlapped among different *dcl2/4* groups (Figure 1d). Comparing enriched GO terms of *dcl2/4* groups, it was found that CMV, CMV-Δ2b and CMV-2aTΔ2b infection in *dcl2/4* elicited an increased expression of the host genes that are related to hypoxia stress (GO:0001666, response to hypoxia; GO:0036293, response to decreased oxygen levels; GO:0070482, response to oxygen levels; GO:0071456, cellular response to hypoxia; GO:0036294, cellular response to decreased oxygen levels; GO:0071453, cellular response to oxygen levels; GO:0006979, response to oxidative stress), immune system to biotic stress (GO:0042742, defense response to bacterium; GO:0002376, immune system process; GO:0006955, immune response; GO:0045087, innate immune response; GO:0009620, response to fungus), response to nitrogen compounds (GO:1901698), and secretory vesicle (GO:0099503) (Figures S4–S6). The transcription of genes involved in photosynthesis or chloroplast structure (GO:0015979, photosynthesis; GO:0006091, the generation of precursor metabolites and energy; GO:0019684, photosynthesis light reaction; GO:0009657, plastid organization; GO:0009658, chloroplast organization GO:0031976, plastid thylakoid; GO:0009534, chloroplast thylakoid; GO:0042651, thylakoid membrane; GO:0034357, photosynthetic membrane; GO:0055035, plastid thylakoid membrane; GO:0009535, chloroplast thylakoid membrane GO:0031977, thylakoid lumen; GO:0042170, plastid membrane; GO:0031969, chloroplast membrane; GO:0010287, plastoglobule; GO:0009521, photosystem; GO:0009543, chloroplast thylakoid lumen), cellular metabolism (GO:0006520, cellular amino acid metabolic process; GO:0044262, cellular carbohydrate metabolic process; GO:1901137, carbohydrate derivative biosynthetic process; GO:0016051, carbohydrate biosynthetic process; GO:1901605, alpha-amino acid metabolic process; GO:0006790, sulfur compound metabolic process), cellular transport (GO:0048046, apoplast; GO:0008509, anion transmembrane transporter activity; GO:0015267, channel activity; GO:0022803, passive transmembrane transporter activity), abiotic stress response (GO:0009409, response to cold; GO:0001101, response to acid chemical) and protein activity (GO:0016829, lyase activity; GO:0016853, isomerase activity) were suppressed in *dcl2/4*-CMV, *dcl2/4*-Δ2b, *dcl2/4*-2aTΔ2b (Figures S5–S7). The function classification of DEGs in virus-infected Col-0 or *dcl2/4* through GO assignment suggested that a systematic infection of CMV- or VSR-deficient CMV would mimic or cause a hypoxia stress and suppress chloroplast organization and function. When antiviral defense was compromised in *dcl2/4* mutant, the virus infection would induce biotic stress response and suppress some cellular metabolism and transport.

### 3.3. Asymptomatic Infections of CMV-Δ2b or CMV-2aTΔ2b in Col-0 Are in Relation to Cell Division and Cellular Metabolism

Infection with intact CMV caused severe disease symptoms in plants. Deletion or interference of the *2b* gene resulted in asymptomatic infections in wild type (Figure 1a) [21,38,39], indicating a defense response in Col-0 restricted pathogenicity of CMV-Δ2b or CMV-2aTΔ2b. Thus, 278 genes synchronously induced and 97 genes concurrently repressed in Col-Δ2b and Col-2aTΔ2b would act as host factors to prevent disease symptoms in the asymptomatic infection (Figure 1d).

**Figure 2:**
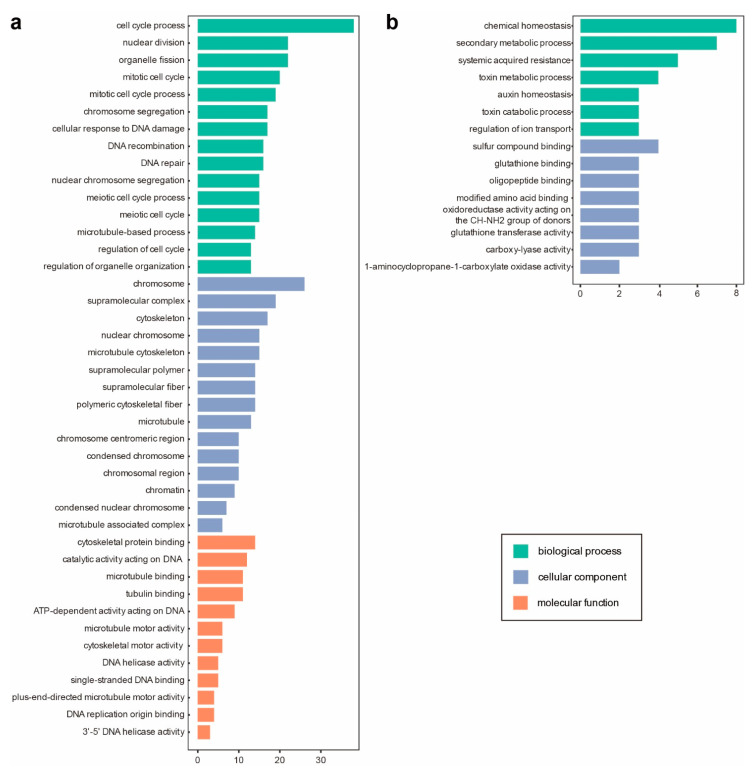
Gene ontology (GO) terms of differentially expressed genes (DEGs) overlapped in asymptomatic infection in Col-Δ2b and Col-2aTΔ2b. (**a**) Top 15 GO terms enriched for induced DEGs. (**b**) GO terms enriched for suppressed DEGs. The y-axis represents the enriched GO term, and the x-axis represents the number of DEGs in the term. Different colors are used to distinguish biological processes, cellular components and molecular functions.

GO assignment was performed to classify gene function of these genes. The three main categories of the GO classification showed that up-regulated genes enriched at fundamental cellular process, including cell cycle (GO:0022402, cell cycle process; GO:0000280, nuclear division, GO:0048285, organelle fission; GO:0000278, mitotic cell cycle; GO:1903047, mitotic cell cycle process; GO:1903046, meiotic cell cycle process; GO:0051321, meiotic cell cycle; GO:0051726, regulation of cell cycle), cytoskeleton component and binding (GO:0007017, microtubule-based process; GO:0099080, supramolecular complex; GO:0005856, cytoskeleton; GO:0015630, microtubule cytoskeleton; GO:0099081, supramolecular polymer; GO:0099512, supramolecular fiber; GO:0099513, polymeric cytoskeletal fiber; GO:0005874, microtubule; GO:0008092, cytoskeletal protein binding; GO:0140097, catalytic activity acting on DNA; GO:0008017, microtubule binding; GO:0015631, tubulin binding), chromosome behavior (GO:0007059, chromosome segregation; GO:0098813, nuclear chromosome segregation; GO:0005694, chromosome; GO:0000228, nuclear chromosome; GO:0000775, chromosome centromeric region; GO:0000793, condensed chromosome; GO:0098687, chromosomal region) and DNA damage and repair (GO:0006974, cellular response to DNA damage stimulus; GO:0006310, DNA recombination; GO:0006281, DNA repair) (Figure 2a), while the most significantly enriched terms of down-regulated genes are involved in metabolic homeostasis (GO:0048878, chemical homeostasis; GO:0019748, secondary metabolic process), and systemic acquired resistance (GO:0009627) (Figure 2b). The higher gene counts and more enriched GO entries of induced DEGs indicated that asymptomatic infections of CMV-Δ2b or CMV-2aTΔ2b triggered expression of cell division related genes and suppressed the expression of genes involved in metabolic homeostasis.

### 3.4. Disease Symptom of Col-CMV, dcl2/4-CMV, dcl2/4-Δ2b, and dcl2/4-2aTΔ2b Could Be Connected to Resistance Genes and Photosynthesis

Except for asymptomatic infection, many virus infections cause host developmental defects, such as CMV (Figure 1a). CMV 2b is not only a VSR, but also a pathogenicity determinant in a CMV–host interaction [27,28]. However, CMV-Δ2b or CMV-2aTΔ2b could induce severe symptoms, such as stunting and curled rosette leaves, in *dcl2/4* and other antiviral RNAi deficient mutants [10,21,31,33,40]. The similar curled systematic infected leaves of Col-CMV, *dcl2/4*-CMV, *dcl2/4*-Δ2b, and *dcl2/4*-2aTΔ2b suggested that disease symptoms in Arabidopsis could also be caused independent of 2b function [26,27].

**Figure 3:**
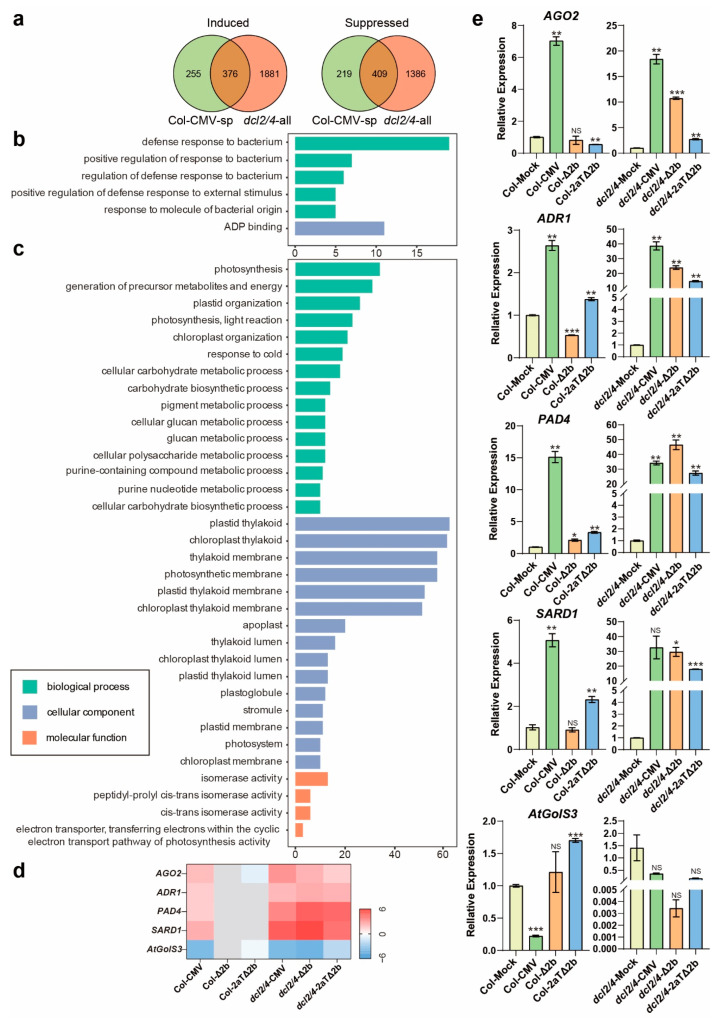
DEGs overlapped in Col-CMV, *dcl2/4*-CMV, *dcl2/4*-Δ2b, and *dcl2/4*-2aTΔ2b. (**a**) Comparison of the number of DEGs among different gene sets in symptomatic infected plants. (**b**) GO terms enriched for induced DEGs. (**c**) Top 15 GO terms enriched for suppressed DEGs. The y-axis represents the enriched GO term, and the x-axis represents the number of DEGs in the term. Different colors are used to distinguish biological processes, cellular components and molecular functions. (**d**) Heat map of part DEGs changed dramatically in sick plants. (**e**) Relative transcriptional level of *AGO2*, *ADR1*, *PAD4*, *SARD1* and *AtGolS3*. Values are means ± SEM. *, *P* < 0.05; **, *P* < 0.01; ***, *P* < 0.001; NS, no significance (*n* = 3, two-tailed Student’s *t*-test).

To discover more host factors involved in symptoms development caused by infection of CMV or CMV mutants, gene classification and function enrichment were performed with DEGs regulated in Col-CMV specifically (Col-CMV-sp) and concurrently mediated in *dcl2/4*-CMV, *dcl2/4*-Δ2b, and *dcl2/4*-2aTΔ2b (*dcl2/4*-all). In Col-0 group, CMV induced 631 genes and suppressed 628 genes specifically (Figure 1c). Meanwhile, *dcl2/4*-CMV, *dcl2/4*-Δ2b, and *dcl2/4*-2aTΔ2b have up-regulated 2257 genes and down-regulated 1795 genes in common (Figure 1c). Comparing DEGs in Col-CMV-sp and *dcl2/4*-all, 376 and 409 genes were concurrently induced or suppressed, respectively, in these four diseased virus-infected plants (Figure 3a).

The 376 induced genes enriched to anti-bacterium immunity (GO:0042742, defense response to bacterium; GO:1900426, the positive regulation of defense response to bacterium; GO:1900424, the regulation of defense response to bacterium; GO:0032103, the positive regulation of response to external stimulus; GO:0002237, response to molecule of bacterial origin) and ADP binding (GO:0043531) (Figure 3b, Table S3). Among all the induced genes that enriched in anti-bacterium immunity related functions, there are genes that have been reported to be involved in antiviral immunity. *AGO2* (AT1G31280), one major antiviral RNAi AGO against RNA virus in Arabidopsis [31,41,42,43,44], is significantly induced in symptomatic plants and maintained at low expression level in healthy Col-0 (Figure 3d,e). Except for the antiviral RNA silencing, resistance (*R*) gene-mediated immunity plays an important role in antiviral defense [45,46,47]. Typical *R* genes, which encode nucleotide-binding, leucine-rich repeat (NB-LRR) proteins, such as *ACTIVATED DISEASE RESISTANCE 1* (*ADR1*, AT1G33560), *PHYTOALEXIN DEFICIENT 4* (*PAD4*, AT3G52430) and *SYSTEMIC ACQUIRED RESISTANCE DEFICIENT 1* (*SARD1*, AT1G73805) were also induced dramatically in diseased plants and only slightly induced in asymptomatic Col-Δ2b and Col-2aTΔ2b (Figure 3d,e). Notably, transcript levels of these four genes increased more dramatically in susceptible *dcl2/4* (Figure 3e). The expression pattern of these four genes is in agreement with the symptom severity of virus-infected plants, suggesting a correlation between the CMV-induced phenotype and the expression of immunity and resistance genes.

On the other hand, the 409 suppressed genes in all symptomatic plants are in association with photosynthesis and chloroplast (GO:0015979, photosynthesis; GO:0006091, the generation of precursor metabolites and energy; GO:0009657, plastid organization; GO:0019684, photosynthesis light reaction; GO:0009658, chloroplast organization; GO:0044262, cellular carbohydrate metabolic process; GO:0016051, carbohydrate biosynthetic process; GO:0006073, cellular glucan metabolic process; GO:0044042, glucan metabolic process; GO:0044264, cellular polysaccharide metabolic process; GO:0034637, cellular carbohydrate biosynthetic process; GO:0031976, plastid thylakoid; GO:0009534, chloroplast thylakoid; GO:0042651, thylakoid membrane; GO:0034357, photosynthetic membrane; GO:0055035, plastid thylakoid membrane; GO:0009535, chloroplast thylakoid membrane; GO:0031977, thylakoid lumen; GO:0009543, chloroplast thylakoid lumen; GO:0031978, plastid thylakoid lumen; GO:0010287, plastoglobule; GO:0010319, stromule; GO:0042170, plastid membrane; GO:0009521, photosystem; GO:0031969, chloroplast membrane; GO:0045156, electron transporter transferring electrons within the cyclic electron transport pathway of photosynthesis activity), cellular metabolism (GO:0042440, pigment metabolic process; GO:0072521, purine-containing compound metabolic process; GO:0006163, purine nucleotide metabolic process), isomerase activity (GO:0016853, isomerase activity; GO:0003755, peptidyl-prolyl cis-trans isomerase activity; GO:0016859, cis-trans isomerase activity;), response to cold (GO:0009409), and apoplast (GO:0048046) (Figure 3c, Table S3). Though the quantity of down-regulated genes is large, few genes are suppressed significantly (Table S3). A putative galactinol synthase gene *GALACTINOL SYNTHASE 3* (*GolS3*, AT1G09350) was suppressed in sick plants (Figure 3d,e). These GO terms suggested a perturbation in photosynthesis and fundamental metabolism in sick plants.

### 3.5. Distinct Pathways Affected in Col-0 Infected by Intact CMV

**Figure 4:**
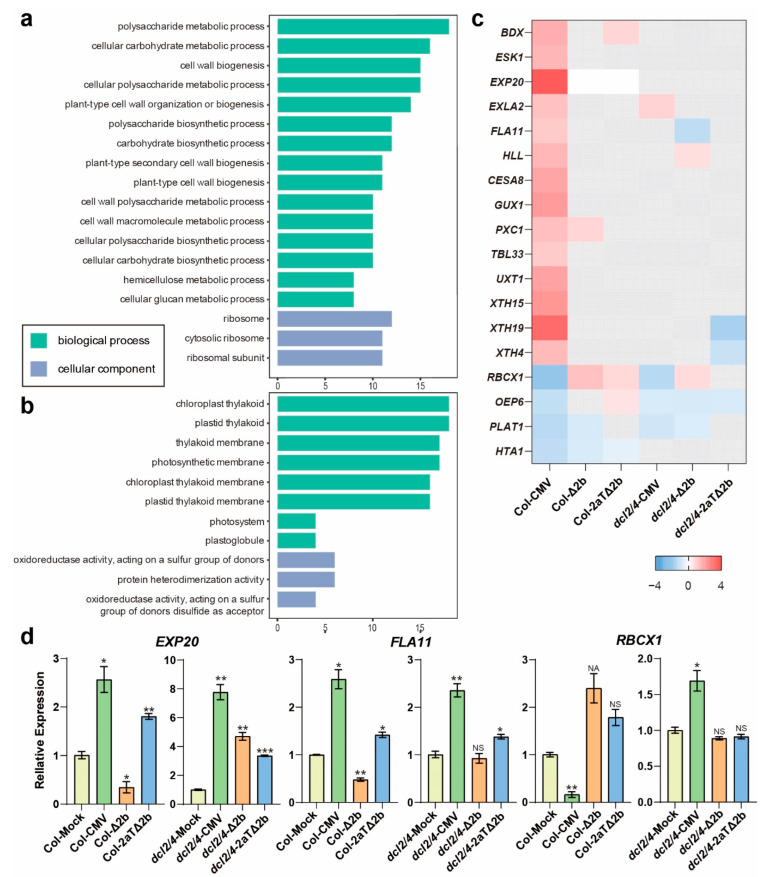
DEGs specifically detected in Col-CMV. (**a**) GO terms enriched for induced DEGs. (**b**) GO terms enriched for suppressed DEGs. The y-axis represents the enriched GO term, and the x-axis represents the number of DEGs in the term. Different colors are used to distinguish biological processes, cellular components and molecular functions. (**c**) Heat map of DEGs enriched in GO terms. (**d**) Relative transcriptional level of *EXP20*, *FLA111*, and *RBCX1*. Values are means ± SEM. *, *P* < 0.05; **, *P* < 0.01; ***, *P* < 0.001; NS, no significance (*n* = 3, two-tailed Student’s *t*-test).

With the removal of DEGs shown in *dcl2/4* group, Col-CMV-sp had other 255 induced DEGs and 219 suppressed DEGs (Figure 3a), which were speculated to be the direct or indirect target of CMV 2b. The GO analysis of DEGs specifically regulated in Col-CMV showed that induced genes are related to polysaccharide metabolism (GO:0005976, polysaccharide metabolic process; GO:0044262, cellular carbohydrate metabolic process; GO:0044264, cellular polysaccharide metabolic process; GO:0000271, polysaccharide biosynthetic process; GO:0016051, carbohydrate biosynthetic process; GO:0010383, cell wall polysaccharide metabolic process; GO:0033692, cellular polysaccharide biosynthetic process; GO:0034637, cellular carbohydrate biosynthetic process; GO:0010410, hemicellulose metabolic process; GO:0006073, cellular glucan metabolic process), cell wall biogenesis (GO:0042546, cell wall biogenesis; GO:0071669, plant-type cell wall organization or biogenesis; GO:0009834, plant-type secondary cell wall biogenesis; GO:0009832, plant-type cell wall biogenesis; GO:0044036, cell wall macromolecule metabolic process) and ribosome (GO:0005840, ribosome; GO:0022626, cytosolic ribosome; GO:0044391, ribosomal subunit) (Figure 4a, Table S4). These GO terms are involved in basic biological processes that are indispensable in cell wall formation. Genes related to cell wall, such as *FASCICLIN-LIKE ARABINOGALACTAN-PROTEIN 11* (*FLA11*, AT5G03170) and *EXPANSIN A20* (*EXPA20*, AT4G38210), were specifically induced in CMV infected Col-0 and *dcl2/4* (Figure 4c,d). The induced DEGs infers that 2b protein may impact the synthesis of polysaccharide and cell wall biogenesis in wild-type Col-0.

Simultaneously, suppressed genes are involved in chloroplast thylakoid (GO:0009534, chloroplast thylakoid; GO:0031976, plastid thylakoid; GO:0042651, thylakoid membrane; GO:0034357, photosynthetic membrane; GO:0009535, chloroplast thylakoid membrane; GO:0055035, plastid thylakoid membrane; GO:0009521, photosystem; GO:0010287, plastoglobule) and protein modification (GO:0016667, oxidoreductase activity acting on a sulfur group of donors; GO:0016671, oxidoreductase activity acting on a sulfur group of donors disulfide as acceptor; GO:0046982, protein heterodimerization activity) (Figure 4b, Table S4). The *HOMOLOGUE OF CYANOBACTERIAL RBCX 1* (*RBCX1*, AT4G04330), which encodes a chloroplast thylakoid localized RbcX protein that acts as a chaperone in the folding of Rubisco [48], was significantly reduced in Col-CMV (Figure 4c,d). These entries would represent the specific genes in photosynthesis suppressed in Col-o after the infection of wild-type CMV.

### 3.6. Pathways Specifically Modified in Antiviral RNAi-Defecient dcl2/4 Mutant

**Figure 5:**
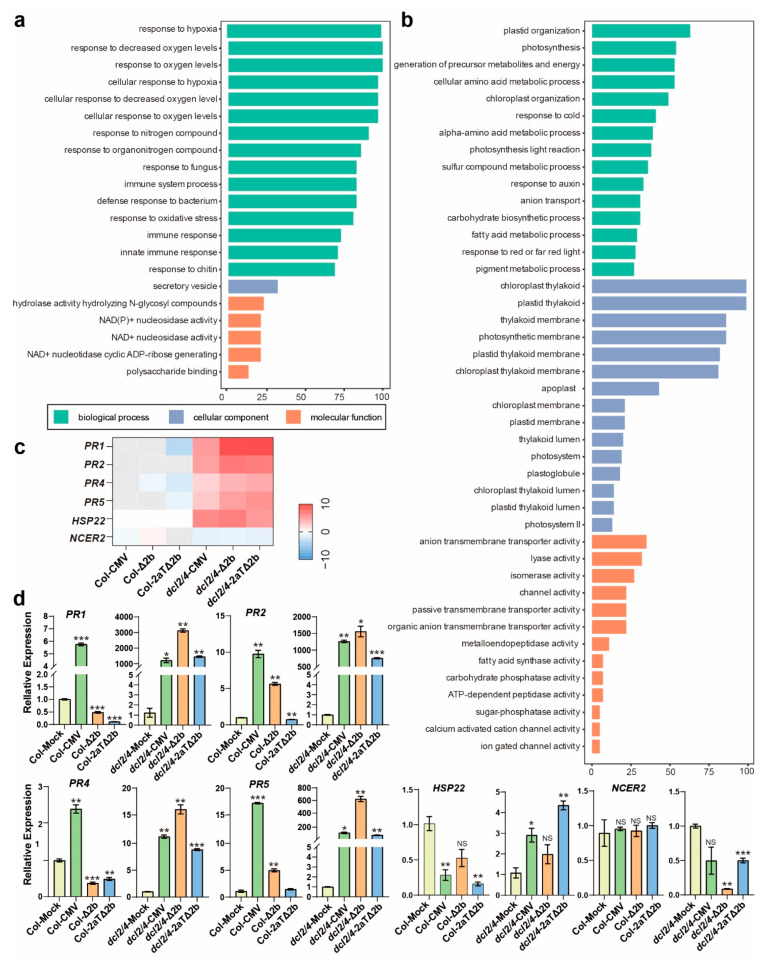
DEGs specifically overlapped in *dcl2/4*-CMV, *dcl2/4*-Δ2b, and *dcl2/4*-2aTΔ2b. (**a**) GO terms enriched for induced DEGs. (**b**) GO terms enriched for suppressed DEGs. The y-axis represents the enriched GO term, and the x-axis represents the number of DEGs in the term. Different colors are used to distinguish biological processes, cellular components and molecular functions. (**c**) Heat map of part DEGs changed dramatically in sick *dcl2/4* plants. (**d**) Relative transcriptional level of *PR1*, *PR2*, *PR4*, *PR5*, *HSP22* and *NCER2*. Values are means ± SEM. *, *P* < 0.05; **, *P* < 0.01; ***, *P* < 0.001; NS, no significance (*n* = 3, two-tailed Student’s *t*-test).

Virus infection on hyper-susceptible *dcl2/4* presumably elicits an antiviral defense that is covered by RNAi silencing in wild type. With a defected antiviral RNAi, virus infection specifically induced 1881 and suppressed 1386 genes in *dcl2/4* (Figure 3a). The GO analysis of DEGs that overlapped in all *dcl2/4* groups but was expressed in a different manner in Col-CMV was performed to investigate putative anti-viral immunity genes. Induced genes only in *dcl2/4* group are associated with hypoxia stress response (GO:0001666, response to hypoxia; GO:0036293, response to decreased oxygen levels; GO:0070482, response to oxygen levels; GO:0071456, cellular response to hypoxia; GO:0036294, cellular response to decreased oxygen levels; GO:0071453, cellular response to oxygen levels; GO:0006979, response to oxidative stress), immune response to pathogens (GO:0002376, immune system process; GO:0006955, immune response; GO:0045087, innate immune response; GO:0009620, response to fungus; GO:0042742, defense response to bacterium; GO:0010200, response to chitin), nitrogen response (GO:1901698, response to nitrogen compound, GO:0010243, response to organonitrogen compound) and NAD metabolism (GO:0050135, NAD(P)^+^ nucleosidase activity; GO:0003953, NAD^+^ nucleosidase activity; GO:0061809, NAD^+^ nucleotidase cyclic ADP-ribose generating) (Figure 5a, Table S5). Pathogen-related genes (PRs), *PR1* (AT2G14610), *PR2* (AT3G57260), *PR4* (AT3G04720) and *PR5* (AT1G75040) were found specially induced in virus infected *dcl2/4* and screened out in Col-CMV with a Padjust-value > 0.5 (Figure 5c). Real-time quantitative PCR showed that *PR* genes induced drastically in virus infected *dcl2/4* and relatively slightly in Col-CMV (Figure 5d). A heat shock protein gene, *HSP22*, was induced specifically in *dcl2/4* (Figure 5d). GO enrichment and transcriptional level showed that abiotic and biotic stress responsive genes were specifically induced in *dcl2/4* after the infection of CMV, CMV-Δ2b or CMV-2aTΔ2b.

In addition, genes only suppressed in *dcl2/4* group are related to photosynthesis and chloroplast organization (GO:0009657, plastid organization; GO:0015979, photosynthesis; GO:0009658, chloroplast organization; GO:0019684, photosynthesis light reaction; GO:0009639, response to red or far red light; GO:0009534, chloroplast thylakoid; GO:0031976, plastid thylakoid; GO:0042651, thylakoid membrane; GO:0034357, photosynthetic membrane; GO:0055035, plastid thylakoid membrane; GO:0009535, chloroplast thylakoid membrane; GO:0031969, chloroplast membrane; GO:0042170, plastid membrane; GO:0031977, thylakoid lumen; GO:0009521, photosystem; GO:0010287, plastoglobule; GO:0009543, chloroplast thylakoid lumen; GO:0031978, plastid thylakoid lumen; GO:0009523, photosystem II), fundamental substance metabolic process (GO:0006091, generation of precursor metabolites and energy; GO:0016051, carbohydrate biosynthetic process; GO:0006520, cellular amino acid metabolic process; GO:1901605, alpha-amino acid metabolic process; GO:0006631, fatty acid metabolic process; GO:0004312, fatty acid synthase activity; GO:0006790, sulfur compound metabolic process; GO:0042440, pigment metabolic process), response to cold (GO:0009409), response to auxin (GO:0009733), ion transport (GO:0048046, apoplast; GO:0006820, anion transport; GO:0008509, anion transmembrane transporter activity; GO:0015267, channel activity; GO:0022803, passive transmembrane transporter activity; GO:0008514, organic anion transmembrane transporter activity; GO:0005227, calcium activated cation channel activity; GO:0022839, ion gated channel activity) and various protein activity (GO:0016829, lyase activity; GO:0016853, isomerase activity; GO:0004222, metalloendopeptidase activity; GO:0004176, ATP-dependent peptidase activity; GO:0019203, carbohydrate phosphatase activity; GO:0050308, sugar-phosphatase activity) (Figure 5b, Table S5). A putative neutral/alkaline non-lysosomal ceramidase, *NEUTRAL CERAMIDASE 2* (*NCER2*, AT2G38010) was significantly reduced in *dcl2/4* (Figure 5d). Thus, multiple aspects of biological pathways could be suppressed in the hyper-susceptible *dcl2/4* after viral infection.

## 4. Discussion

Transcriptome analysis offers a general view of affected pathways and development in virus-infected plants. Systematical infection with CMV or CMV mutants brings about some common physiological changes in host plants. Previous studies revealed that the virus infection can modify photosynthesis and disturb chloroplast components and functions [49,50,51,52], and coat protein of CMV is responsible for the chlorosis in infected leaves [51]. Indeed, we found numerous genes implicated in photosynthesis and chloroplast organization reduced expression pattern in CMV-infected plants (Figures S2–S7). In addition, genes involved in cold response are also suppressed in all the six CMV infected plants (Figures S2–S7). Considering that virus-triggered RNA silencing is inhibited at low temperature and CMV-infected plants increases tolerance to frost stress [53,54], there should be a crosstalk between cold response and virus infection.

In this work, genes related to hypoxia response are also consistently induced in all infected plants (Figures S2–S7), which is manifested by the waterlogging phenotype of the systematically infected leaves (Figure 1a). The hypoxia response in systematically infected leaves may be due to the disturbance on the photoreaction and respiratory electron transport by virus infection [55]. It has been found that hypoxia tolerance required AGO1 and AGO4 RNA-silencing pathways [56,57,58,59], while virus infection silenced AGO1 [60]. Thus, the host plant may elevate hypoxia response to restore hypoxia tolerance upon viral infection.

As for the asymptomatic infection of CMV-Δ2b or CMV-2aTΔ2b to Col-0, lots of cell division related genes increased expression and genes involved in chemical homeostasis and acquired resistance were inhibited (Figure 2). We speculate that host plants reduce resistance and viruses promote cell division in hosts during the asymptomatic virus infection to achieve the mutualistic interaction between CMV-Δ2b or CMV-2aTΔ2b and Col-0. However, in symptomatic infected plants, bacterium immunity is elevated and cellular metabolism was reduced (Figure 3). The accumulation of *AGO2* mRNA may be induced by the reduction of miR403 (Figure 3d, e) [61], which depends on the suppression of the AGO1 function by virus infection [60]. Enhanced AGO2 activation accompanies induced resistance to CMV in Arabidopsis [42,61,62]. Besides, SA-dependent defense is an effective antiviral strategy and responsible for the virus-caused symptoms [63]. Critical SA-related genes, PAD4, ADR1, and SARD1, are induced in the four diseased plants (Figure 3d,e). EDS1/PAD4 pathway promotes salicylic acid (SA) biosynthesis and maintains important SA-related resistance programs [64]. Activated by both surface-resident and intracellular LRR receptors, EDS1–PAD4–ADR1 signaling pathway acts as the convergence point for defense signaling cascades in pathogen immunity [65]. ADR1 exhibits resistance to a number of microbial pathogens, including CMV [66]. SARD1 is a key regulator for Isochorismate Synthase 1 induction and salicylic acid (SA) synthesis [67]. These induced resistance pathways may contribute to host survival upon virus aggression, and synchronously lead to the development of symptoms.

In modestly sick Col-0 infected with CMV, the cell wall component synthesis and cell wall related genes, FLA11 and EXPA20, which are associated with modification of cell wall [68,69], are specifically upregulated (Figure 4a,c,d). Regarded as a specific response to viruses, cell wall reorganization affects the spread of the virus by involving apoplast and symplast activation [70]. FLA11 and EXPA20 may take part in the cell wall reorganization in CMV-infected Arabidopsis in nature. The increased cell wall biogenesis may attenuate the developmental anomalies in Col-CMV, by contrast to the severely diseased phenotype of *dcl2/4*-CMV, *dcl2/4*-Δ2b and *dcl2/4*-2aTΔ2b.

PR proteins play important roles in response to abiotic and biotic stresses. Usually, the expressions of *PRs* are induced in systemic acquired resistance (SAR) or local acquired resistance (LCR) and minimize the accumulation of pathogens in uninfected plants organs [71,72,73,74]. Interestingly, the expression of *PR1*, *PR2*, *PR4* and *PR5* were increased drastically in virus-infected *dcl2/4* but induced in a relative low extent in Col-CMV, and even reduced in Col-Δ2b and Col-2aTΔ2b (Figure 5d). The highest levels of *PRs* in *dcl2/4-*Δ2b indicated the extreme induction of SA signaling, which should require pathogenicity but is independent of VSR 2b, in the RNAi-deficient plant. In addition, consistent with our above findings, the increased SA-mediated resistance would be related to the severe disease symptom in virus-infected *dcl2/4* and could contribute to a major immunity for *dcl2/4* survival under virus attack. Evidence also showed that disrupting *NCER2* would induce higher levels of SA [75] and loss of *NCER2* function can reduce Arabidopsis resistant to pathogens [76]. Indeed, the level of *NCER2* significantly reduced in *dcl2/4* and *dcl2/4* was more susceptive to CMV compared with Col-0.

In summary, by comparing transcriptome sequence, we found that the systematic infection of CMV, CMV-Δ2b and CMV-2aTΔ2b cause hypoxia response and reduce photosynthesis. Cell wall organization and biogenesis-related genes are specifically induced in asymptomatic infected Col-Δ2b and Col-2aTΔ2b, which may facilitate the symbiosis of CMV mutants and Col-0. The symptom of Col-CMV, *dcl2/4*-CMV, *dcl2/4*-Δ2b and *dcl2/4*-2aTΔ2b is in correlation with the increased AGO2 and pathogen immunity. In addition, cell wall reorganization caused by CMV may relieve symptom development. Nevertheless, the strong activation of SA signaling might lead to severe symptoms in *dcl2/4*. Our research further revealed the interaction between plant Arabidopsis and CMV and provided clues in symptom development and antiviral defenses in plants. Further studies on the response of plant mutants in these biological processes would help to clarify and elucidate the mechanism underlying.

## Data Availability

All of the detailed information regarding the quality assessment, regression analysis and program commands used in Stata software are available in the Supplementary Materials.

## Supplementary Materials

The following supporting information can be downloaded at: https://www.mdpi.com/article/10.3390/v14071582/s1, Figure S1: Principal component analysis (PCA) of biological triplicates derived from RNA-seq; Figure S2: Gene ontology (GO) terms of differentially expressed genes (DEGs) in Col-CMV; Figure S3. GO terms of DEGs in Col-Δ2b; Figure S4. GO terms of DEGs in Col-2aTΔ2b; Figure S5. GO terms of DEGs in *dcl2/4*-CMV; Figure S6. GO terms of DEGs in *dcl2/4*-Δ2b; Figure S7. GO terms of DEGs *dcl2/4*-2aTΔ2b; Table S1. Primers used for Qpcr; Table S2. Analysis of transcriptome sequencing data of Arabidopsis-infected CMV; Table S3. The log_2_Foldchange of DEGs in all diseased plants; Table S4. The log_2_Foldchange of DEGs specifically detected in Col-CMV; Table S5. The log_2_Foldchange of DEGs specifically detected in all virus-infected *dcl2/4*.

## References

[1] Savary S., Willocquet L., Pethybridge S.J., Esker P., McRoberts N., Nelson A. (2019). The global burden of pathogens and pests on major food crops. Nat. Ecol. Evol..

[2] Scholthof K.B., Adkins S., Czosnek H., Palukaitis P., Jacquot E., Hohn T., Hohn B., Saunders K., Candresse T., Ahlquist P. (2011). Top 10 plant viruses in molecular plant pathology. Mol. Plant Pathol..

[3] Pagan I., Fraile A., Fernandez-Fueyo E., Montes N., Alonso-Blanco C., Garcia-Arenal F. (2010). *Arabidopsis thaliana* as a model for the study of plant-virus co-evolution. Philos. Trans. R. Soc. B.

[4] Camborde L., Planchais S., Tournier V., Jakubiec A., Drugeon G., Lacassagne E., Pflieger S., Chenon M., Jupin I. (2010). The ubiquitin-proteasome system regulates the accumulation of *Turnip yellow mosaic virus* RNA-dependent RNA polymerase during viral infection. Plant Cell.

[5] Diaz-Pendon J.A., Li F., Li W.X., Ding S.W. (2007). Suppression of antiviral silencing by cucumber mosaic virus 2b protein in *Arabidopsis* is associated with drastically reduced accumulation of three classes of viral small interfering RNAs. Plant Cell.

[6] Elena S.F., Rodrigo G. (2012). Towards an integrated molecular model of plant-virus interactions. Curr. Opin. Virol..

[7] Garcia D., Garcia S., Voinnet O. (2014). Nonsense-mediated decay serves as a general viral restriction mechanism in plants. Cell Host Microbe.

[8] Garcia-Ruiz H. (2019). Host factors against plant viruses. Mol. Plant Pathol..

[9] Garcia-Ruiz H., Takeda A., Chapman E.J., Sullivan C.M., Fahlgren N., Brempelis K.J., Carrington J.C. (2010). *Arabidopsis* RNA-dependent RNA polymerases and Dicer-like proteins in antiviral defense and small interfering RNA biogenesis during *Turnip Mosaic Virus* infection. Plant Cell.

[10] Guo Z., Lu J., Wang X., Zhan B., Li W., Ding S.W. (2017). Lipid flippases promote antiviral silencing and the biogenesis of viral and host siRNAs in Arabidopsis. Proc. Natl. Acad. Sci. USA.

[11] Lellis A.D., Kasschau K.D., Whitham S.A., Carrington J.C. (2002). Loss-of-susceptibility mutants of *Arabidopsis thaliana* reveal an essential role for eIF(iso)4E during potyvirus infection. Curr. Biol..

[12] Li F.F., Zhang C.W., Li Y.Z., Wu G.W., Hou X.L., Zhou X.P., Wang A.M. (2018). Beclin1 restricts RNA virus infection in plants through suppression and degradation of the viral polymerase. Nat. Commun..

[13] Yoshii M., Nishikiori M., Tomita K., Yoshioka N., Kozuka R., Naito S., Ishikawa M. (2004). The *Arabidopsis cucumovirus multiplication* 1 and 2 loci encode translation initiation factors 4E and 4G. J. Virol..

[14] Ding S.W. (2010). RNA-based antiviral immunity. Nat. Rev. Immunol..

[15] Guo Z., Li Y., Ding S.W. (2019). Small RNA-based antimicrobial immunity. Nat. Rev. Immunol..

[16] Yang Z., Li Y. (2018). Dissection of RNAi-based antiviral immunity in plants. Curr. Opin. Virol..

[17] Blevins T., Rajeswaran R., Shivaprasad P.V., Beknazariants D., Si-Ammour A., Park H.S., Vazquez F., Robertson D., Meins F., Hohn T. (2006). Four plant Dicers mediate viral small RNA biogenesis and DNA virus induced silencing. Nucleic Acids Res..

[18] Bouche N., Lauressergues D., Gasciolli V., Vaucheret H. (2006). An antagonistic function for *Arabidopsis* DCL2 in development and a new function for DCL4 in generating viral siRNAs. EMBO J..

[19] Fukudome A., Fukuhara T. (2017). Plant dicer-like proteins: Double-stranded RNA-cleaving enzymes for small RNA biogenesis. J. Plant Res..

[20] Qu F., Ye X., Morris T.J. (2008). *Arabidopsis* DRB4, AGO1, AGO7, and RDR6 participate in a DCL4-initiated antiviral RNA silencing pathway negatively regulated by DCL1. Proc. Natl. Acad. Sci. USA.

[21] Wang X.B., Wu Q., Ito T., Cillo F., Li W.X., Chen X., Yu J.L., Ding S.W. (2010). RNAi-mediated viral immunity requires amplification of virus-derived siRNAs in *Arabidopsis thaliana*. Proc. Natl. Acad. Sci. USA.

[22] Deleris A., Gallego-Bartolome J., Bao J., Kasschau K.D., Carrington J.C., Voinnet O. (2006). Hierarchical action and inhibition of plant Dicer-like proteins in antiviral defense. Science.

[23] Jin Y., Zhao J.H., Guo H.S. (2020). Recent advances in understanding plant antiviral RNAi and viral suppressors of RNAi. Curr. Opin. Virol..

[24] Duan C.G., Fang Y.Y., Zhou B.J., Zhao J.H., Hou W.N., Zhu H., Ding S.W., Guo H.S. (2012). Suppression of *Arabidopsis* ARGONAUTE1-mediated slicing, transgene-induced RNA silencing, and DNA methylation by distinct domains of the *Cucumber mosaic virus* 2b protein. Plant Cell.

[25] Fang Y.Y., Zhao J.H., Liu S.W., Wang S., Duan C.G., Guo H.S. (2016). CMV2b-AGO interaction is required for the suppression of RDR-Dependent antiviral silencing in *Arabidopsis*. Front Microbiol..

[26] Hamera S., Song X., Su L., Chen X., Fang R. (2012). Cucumber mosaic virus suppressor 2b binds to AGO4-related small RNAs and impairs AGO4 activities. Plant J..

[27] Zhang X.R., Yuan Y.R., Pei Y., Lin S.S., Tuschl T., Patel D.J., Chua N.H. (2006). Cucumber mosaic virus-encoded 2b suppressor inhibits *Arabidopsis* Argonaute1 cleavage activity to counter plant defense. Gene Dev..

[28] Ding S.-W., Li W.-X., Symons R. (1995). A novel naturally occurring hybrid gene encoded by a plant RNA virus facilitates long distance virus movement. EMBO J..

[29] Du Z.Y., Chen F.F., Liao Q.S., Zhang H.R., Chen Y.F., Chen J.S. (2007). 2b ORFs encoded by subgroup IB strains of cucumber mosaic virus induce differential virulence on *Nicotiana* species. J. Gen. Virol..

[30] Soards A.J., Murphy A.M., Palukaitis P., Carr J.P. (2002). Virulence and differential local and systemic spread of *Cucumber mosaic virus* in tobacco are affected by the CMV 2b protein. Mol. Plant Microbe Interact..

[31] Wang X.-B., Jovel J., Udomporn P., Wang Y., Wu Q., Li W.-X., Gasciolli V., Vaucheret H., Ding S.-W. (2011). The 21-nucleotide, but not 22-nucleotide, viral secondary small interfering rnas direct potent antiviral defense by two cooperative argonautes in *Arabidopsis thaliana*. Plant Cell.

[32] Gao H., Yang M., Yang H., Qin Y., Zhu B., Xu G., Xie C., Wu D., Zhang X., Li W. (2018). Arabidopsis ENOR3 regulates RNAi-mediated antiviral defense. J. Genet. Genom..

[33] Guo Z., Wang X.B., Wang Y., Li W.X., Gal-On A., Ding S.W. (2018). Identification of a new host factor required for antiviral RNAi and amplification of viral siRNAs. Plant Physiol..

[34] Le S., Josse J., Husson F. (2008). FactoMineR: An R package for multivariate analysis. J. Stat. Softw..

[35] Love M., Anders S., Huber W. (2014). Differential analysis of count data–the DESeq2 package. Genome Biol..

[36] Yu G., Wang L.-G., Han Y., He Q.-Y. (2012). clusterProfiler: An R package for comparing biological themes among gene clusters. Omics J. Integr. Biol..

[37] Livak K.J., Schmittgen T.D. (2001). Analysis of relative gene expression data using real-time quantitative PCR and the 2^−ΔΔCT^ method. Methods.

[38] Lewsey M., Surette M., Robertson F.C., Ziebell H., Choi S.H., Ryu K.H., Canto T., Palukaitis P., Payne T., Walsh J.A. (2009). The role of the *Cucumber mosaic virus* 2b protein in viral movement and symptom induction. Mol. Plant Microbe Interact..

[39] Hou W.N., Duan C.G., Fang R.X., Zhou X.Y., Guo H.S. (2011). Satellite RNA reduces expression of the 2b suppressor protein resulting in the attenuation of symptoms caused by *Cucumber mosaic virus* infection. Mol. Plant Pathol..

[40] Zhu B., Gao H., Xu G., Wu D., Song S., Jiang H., Zhu S., Qi T., Xie D. (2017). Arabidopsis ALA1 and ALA2 mediate RNAi-based antiviral immunity. Front. Plant Sci..

[41] Garcia-Ruiz H., Carbonell A., Hoyer J.S., Fahlgren N., Gilbert K.B., Takeda A., Giampetruzzi A., Garcia Ruiz M.T., McGinn M.G., Lowery N. (2015). Roles and programming of Arabidopsis ARGONAUTE proteins during *Turnip mosaic virus* infection. PLoS Pathog..

[42] Harvey J.J., Lewsey M.G., Patel K., Westwood J., Heimstadt S., Carr J.P., Baulcombe D.C. (2011). An antiviral defense role of AGO2 in plants. PLoS ONE.

[43] Jaubert M., Bhattacharjee S., Mello A.F., Perry K.L., Moffett P. (2011). ARGONAUTE2 mediates RNA-silencing antiviral defenses against Potato virus X in Arabidopsis. Plant Physiol..

[44] Zhang X., Zhang X., Singh J., Li D., Qu F. (2012). Temperature-dependent survival of *Turnip crinkle virus*-infected arabidopsis plants relies on an RNA silencing-based defense that requires DCL2, AGO2, and HEN1. J. Virol..

[45] Ando S., Miyashita S., Takahashi H. (2019). Plant defense systems against cucumber mosaic virus: Lessons learned from CMV-Arabidopsis interactions. J. Gen. Plant Pathol..

[46] De Ronde D., Butterbach P., Kormelink R. (2014). Dominant resistance against plant viruses. Front. Plant Sci..

[47] Mandadi K.K., Scholthof K.B. (2013). Plant immune responses against viruses: How does a virus cause disease?. Plant Cell.

[48] Zhang R., Zhi H., Li Y., Guo E., Feng G., Tang S., Guo W., Zhang L., Jia G., Diao X. (2022). Response of Multiple Tissues to Drought Revealed by a Weighted Gene Co-Expression Network Analysis in Foxtail Millet [*Setaria italica* (L.) P. Beauv.]. Front. Plant Sci..

[49] Kyselakova H., Prokopova J., Naus J., Novak O., Navratil M., Safarova D., Spundova M., Ilik P. (2011). Photosynthetic alterations of pea leaves infected systemically by pea enation mosaic virus: A coordinated decrease in efficiencies of CO_2_ assimilation and photosystem II photochemistry. Plant Physiol. Biochem..

[50] Liu Y., Liu Y., Spetz C., Li L., Wang X. (2020). Comparative transcriptome analysis in Triticum aestivum infecting wheat dwarf virus reveals the effects of viral infection on phytohormone and photosynthesis metabolism pathways. Phytopathol. Res..

[51] Mochizuki T., Yamazaki R., Wada T., Ohki S.T. (2014). Coat protein mutations in an attenuated *Cucumber mosaic virus* encoding mutant 2b protein that lacks RNA silencing suppressor activity induces chlorosis with photosynthesis gene repression and chloroplast abnormalities in infected tobacco plants. Virology.

[52] Técsi L.I., Maule A.J., Smith A.M., Leegood R.C. (1994). Complex, localized changes in CO_2_ assimilation and starch content associated with the susceptible interaction between cucumber mosaic virus and a cucurbit host. Plant J..

[53] Szittya G., Silhavy D., Molnar A., Havelda Z., Lovas A., Lakatos L., Banfalvi Z., Burgyan J. (2003). Low temperature inhibits RNA silencing-mediated defence by the control of siRNA generation. EMBO J..

[54] Xu P., Chen F., Mannas J.P., Feldman T., Sumner L.W., Roossinck M.J. (2008). Virus infection improves drought tolerance. New Phytol..

[55] Song X.S., Wang Y.J., Mao W.H., Shi K., Zhou Y.H., Nogués S., Yu J.Q. (2009). Effects of cucumber mosaic virus infection on electron transport and antioxidant system in chloroplasts and mitochondria of cucumber and tomato leaves. Physiol. Plant.

[56] Loreti E., Betti F., Ladera-Carmona M.J., Fontana F., Novi G., Valeri M.C., Perata P. (2020). ARGONAUTE1 and ARGONAUTE4 regulate gene expression and hypoxia tolerance. Plant Physiol..

[57] Licausi F., Weits D.A., Pant B.D., Scheible W.R., Geigenberger P., van Dongen J.T. (2011). Hypoxia responsive gene expression is mediated by various subsets of transcription factors and miRNAs that are determined by the actual oxygen availability. New Phytol..

[58] Moldovan D., Spriggs A., Yang J., Pogson B.J., Dennis E.S., Wilson I.W. (2010). Hypoxia-responsive microRNAs and trans-acting small interfering RNAs in Arabidopsis. J. Exp. Bot..

[59] Zhai L.H., Liu Z.J., Zou X.L., Jiang Y.Y., Qiu F.Z., Zheng Y.L., Zhang Z.X. (2013). Genome-wide identification and analysis of microRNA responding to long-term waterlogging in crown roots of maize seedlings. Physiol. Plant..

[60] Varallyay E., Valoczi A., Agyi A., Burgyan J., Havelda Z. (2010). Plant virus-mediated induction of miR168 is associated with repression of ARGONAUTE1 accumulation. EMBO J..

[61] Lewsey M.G., Murphy A.M., Maclean D., Dalchau N., Westwood J.H., Macaulay K., Bennett M.H., Moulin M., Hanke D.E., Powell G. (2010). Disruption of two defensive signaling pathways by a viral RNA silencing suppressor. Mol. Plant Microbe Interact..

[62] Ando S., Jaskiewicz M., Mochizuki S., Koseki S., Miyashita S., Takahashi H., Conrath U. (2021). Priming for enhanced ARGONAUTE2 activation accompanies induced resistance to cucumber mosaic virus in *Arabidopsis thaliana*. Mol. Plant Pathol..

[63] Alvarez M.E. (2000). Salicylic acid in the machinery of hypersensitive cell death and disease resistance. Plant Mol. Biol..

[64] Cui H.T., Gobbato E., Kracher B., Qiu J.D., Bautor J., Parker J.E. (2017). A core function of EDS1 with PAD4 is to protect the salicylic acid defense sector in Arabidopsis immunity. New Phytol..

[65] Pruitt R.N., Locci F., Wanke F., Zhang L., Saile S.C., Joe A., Karelina D., Hua C., Frohlich K., Wan W.L. (2021). The EDS1-PAD4-ADR1 node mediates Arabidopsis pattern-triggered immunity. Nature.

[66] Grant J.J., Chini A., Basu D., Loake G.J. (2003). Targeted activation tagging of the Arabidopsis NBS-LRR gene, ADR1, conveys resistance to virulent pathogens. Mol. Plant-Microbe Interact..

[67] Zhang Y., Xu S., Ding P., Wang D., Cheng Y.T., He J., Gao M., Xu F., Li Y., Zhu Z. (2010). Control of salicylic acid synthesis and systemic acquired resistance by two members of a plant-specific family of transcription factors. Proc. Natl. Acad. Sci. USA.

[68] Ma Y., MacMillan C.P., de Vries L., Mansfield S.D., Hao P., Ratcliffe J., Bacic A., Johnson K.L. (2022). FLA11 and FLA12 glycoproteins fine-tune stem secondary wall properties in response to mechanical stresses. New Phytol.

[69] Wang Z., Wang M., Yang C., Zhao L., Qin G., Peng L., Zheng Q., Nie W., Song C., Shi H. (2021). SWO1 modulates cell wall integrity under salt stress by interacting with importin α in Arabidopsis. Stress Biol..

[70] Otulak-Koziel K., Koziel E., Lockhart B.E.L. (2018). Plant cell wall dynamics in compatible and incompatible potato response to infection caused by *Potato Virus Y* (PVY^NTN^). Int. J. Mol. Sci..

[71] Zribi I., Ghorbel M., Brini F. (2021). Pathogenesis Related Proteins (PRs): From Cellular Mechanisms to Plant Defense. Curr. Protein Pept. Sci..

[72] Hamamouch N., Li C., Seo P.J., Park C.M., Davis E.L. (2011). Expression of Arabidopsis pathogenesis-related genes during nematode infection. Mol. Plant Pathol..

[73] Anisimova O.K., Shchennikova A.V., Kochieva E.Z., Filyushin M.A. (2021). Pathogenesis-Related Genes of PR1, PR2, PR4, and PR5 Families Are Involved in the Response to Fusarium Infection in Garlic (*Allium sativum* L.). Int. J. Mol. Sci..

[74] Ali S., Ganai B.A., Kamili A.N., Bhat A.A., Mir Z.A., Bhat J.A., Tyagi A., Islam S.T., Mushtaq M., Yadav P. (2018). Pathogenesis-related proteins and peptides as promising tools for engineering plants with multiple stress tolerance. Microbiol. Res..

[75] Zienkiewicz A., Gömann J., König S., Herrfurth C., Liu Y.T., Meldau D., Feussner I. (2020). Disruption of Arabidopsis neutral ceramidases 1 and 2 results in specific sphingolipid imbalances triggering different phytohormone-dependent plant cell death programmes. New Phytol..

[76] Kato H., Nemoto K., Shimizu M., Abe A., Asai S., Ishihama N., Matsuoka S., Daimon T., Ojika M., Kawakita K. (2022). Recognition of pathogen-derived sphingolipids in Arabidopsis. Science.

