# Supplementary material for: Comparative Transcriptome Analysis of CMV or 2b-Deficient CMV-Infected dcl2dcl4 Reveals the Effects of Viral Infection on Symptom Induction in Arabidopsis thaliana
Source: Viruses. 2022 Jul 21;14(7):1582. doi: 10.3390/v14071582 (PMC9320214; doi:10.3390/v14071582)
Supplement: Supplementary file 1 [file viruses-14-01582-s001.zip › Supplemental Figures.pptx]

## Slide 1
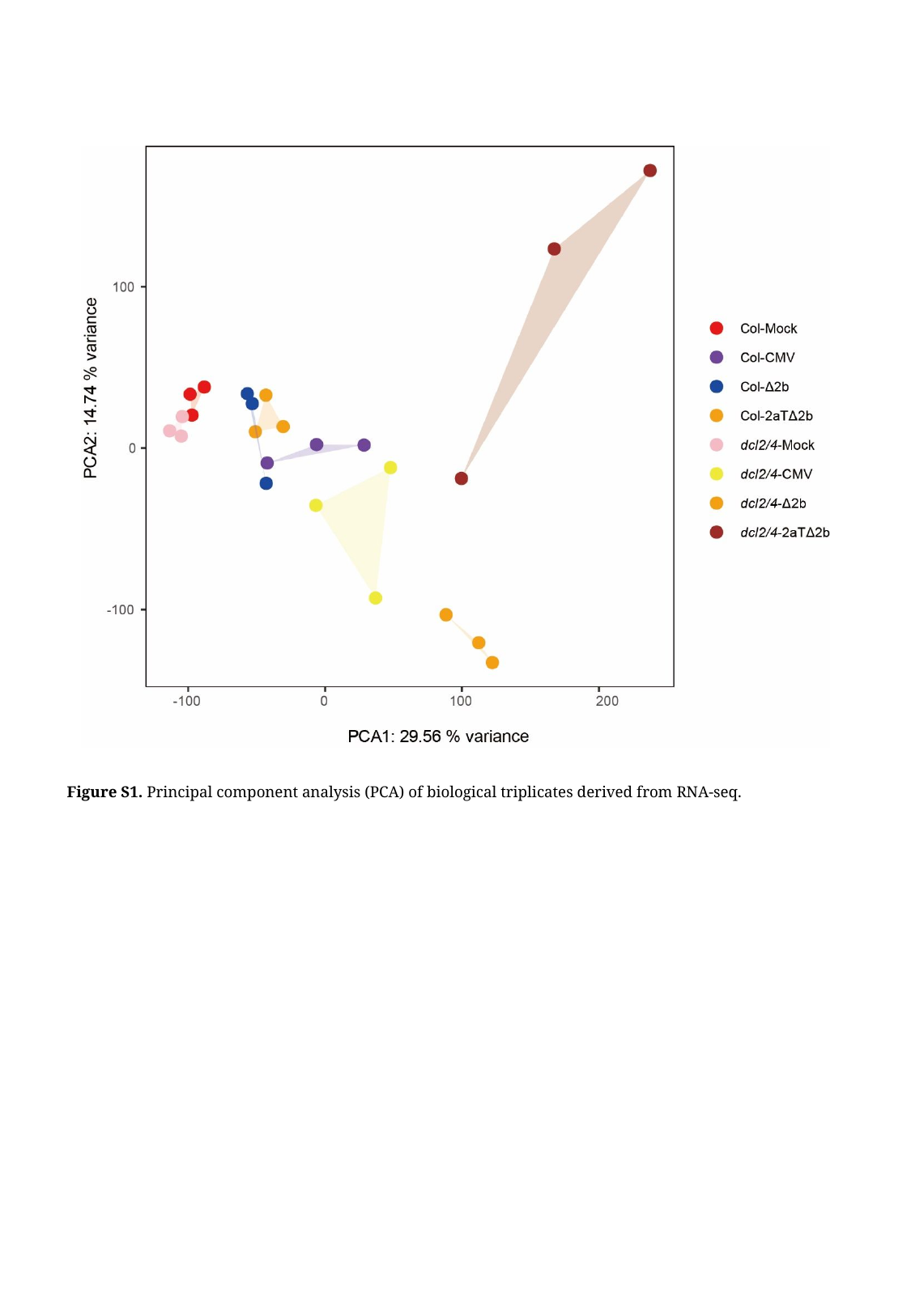

Figure S1. Principal component analysis (PCA) of biological triplicates derived from RNA-seq.

## Slide 2
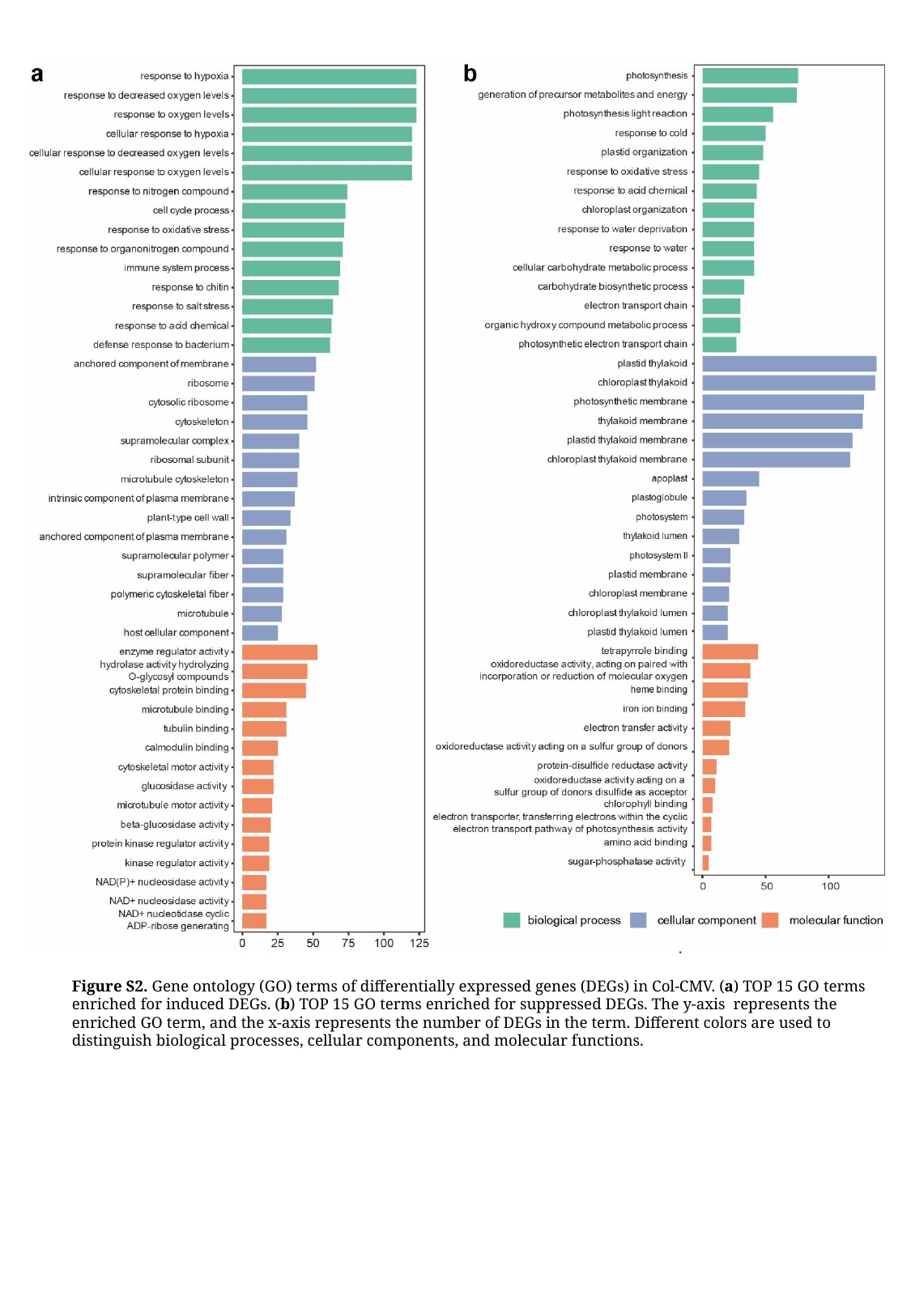

Figure S2. Gene ontology (GO) terms of differentially expressed genes (DEGs) in Col-CMV. (a) TOP 15 GO terms enriched for induced DEGs. (b) TOP 15 GO terms enriched for suppressed DEGs. The y-axis represents the enriched GO term, and the x-axis represents the number of DEGs in the term. Different colors are used to distinguish biological processes, cellular components, and molecular functions.

## Slide 3
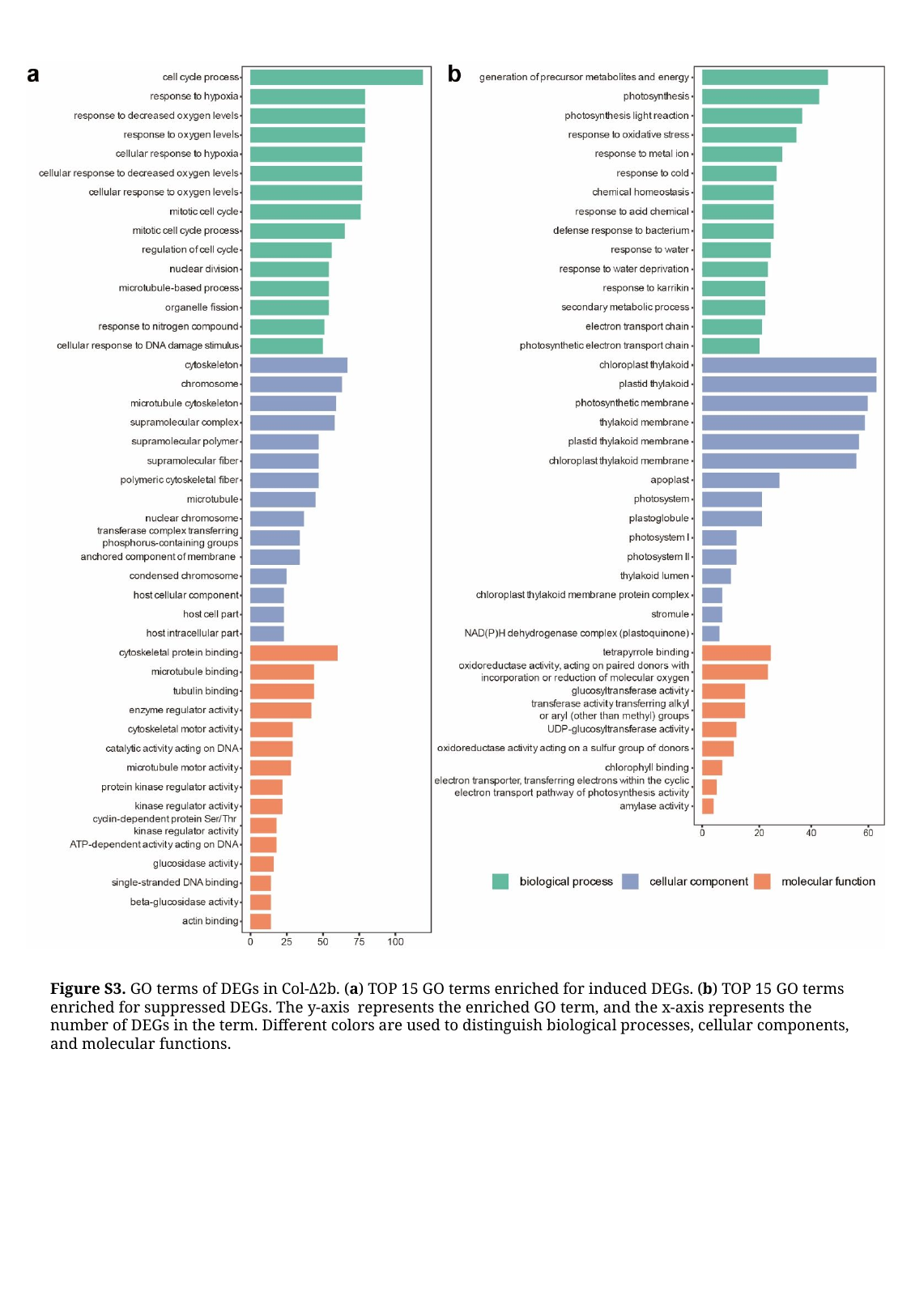

Figure S3. GO terms of DEGs in Col-Δ2b. (a) TOP 15 GO terms enriched for induced DEGs. (b) TOP 15 GO terms enriched for suppressed DEGs. The y-axis represents the enriched GO term, and the x-axis represents the number of DEGs in the term. Different colors are used to distinguish biological processes, cellular components, and molecular functions.

## Slide 4
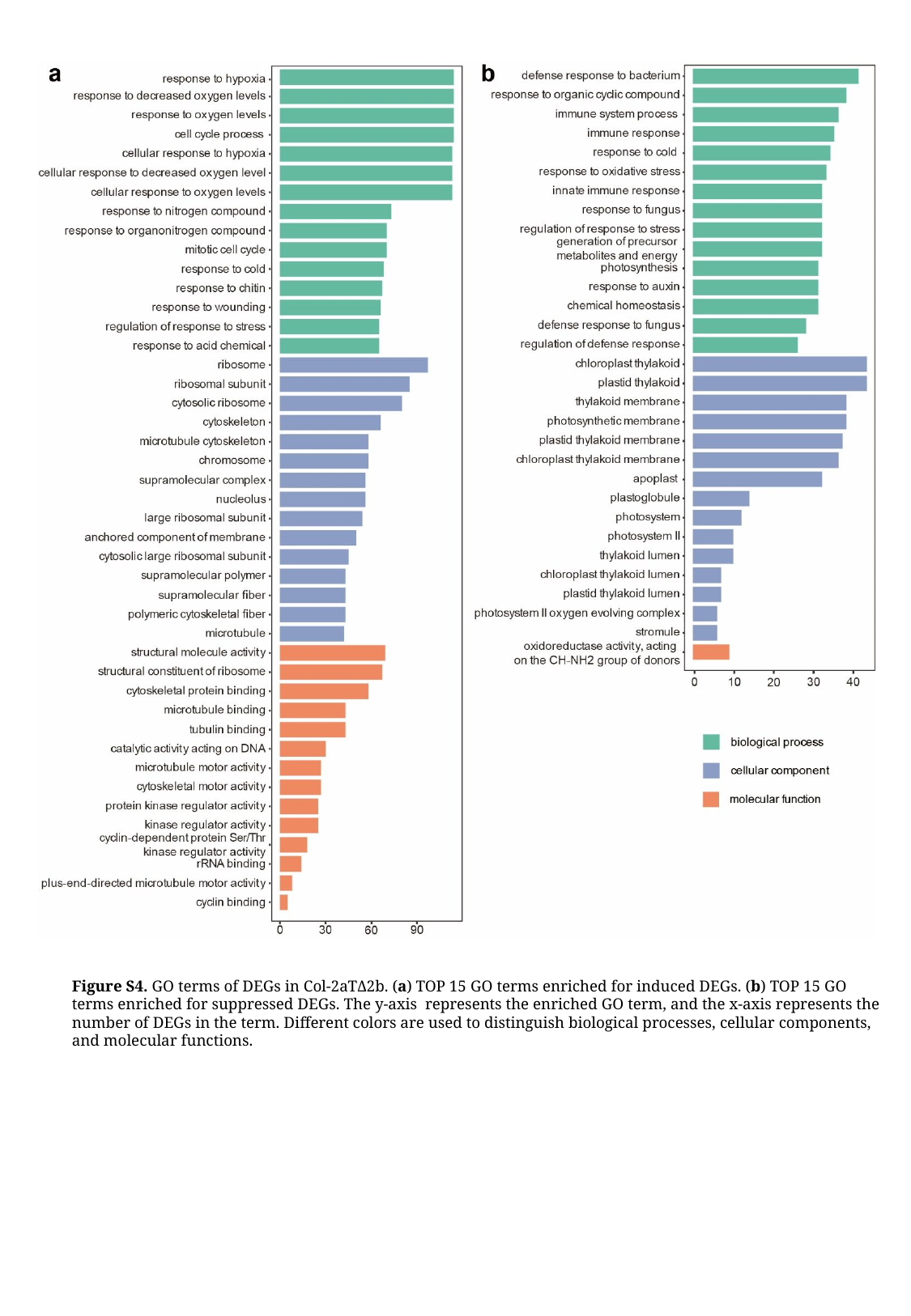

Figure S4. GO terms of DEGs in Col-2aTΔ2b. (a) TOP 15 GO terms enriched for induced DEGs. (b) TOP 15 GO terms enriched for suppressed DEGs. The y-axis represents the enriched GO term, and the x-axis represents the number of DEGs in the term. Different colors are used to distinguish biological processes, cellular components, and molecular functions.

## Slide 5
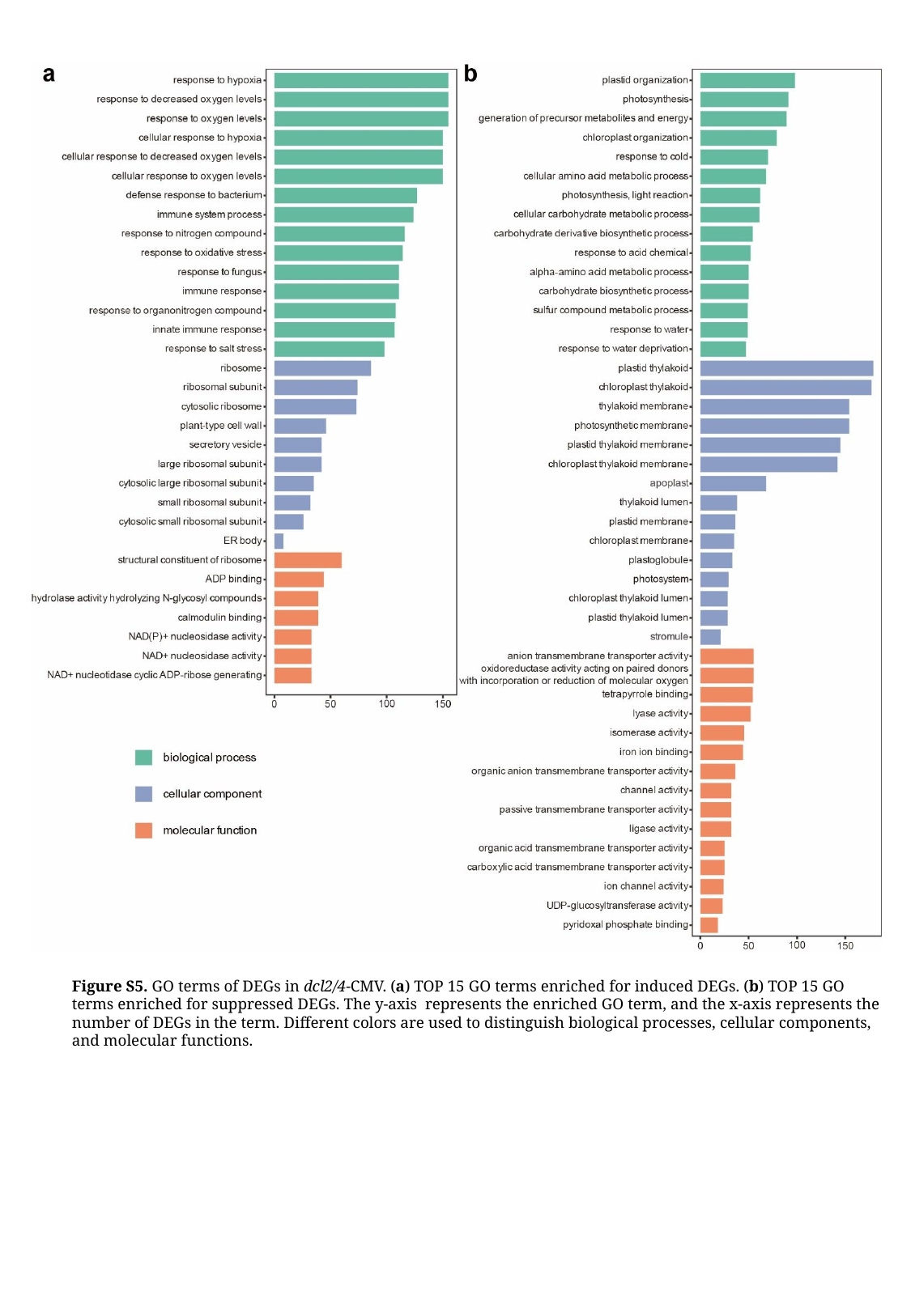

Figure S5. GO terms of DEGs in dcl2/4-CMV. (a) TOP 15 GO terms enriched for induced DEGs. (b) TOP 15 GO terms enriched for suppressed DEGs. The y-axis represents the enriched GO term, and the x-axis represents the number of DEGs in the term. Different colors are used to distinguish biological processes, cellular components, and molecular functions.

## Slide 6
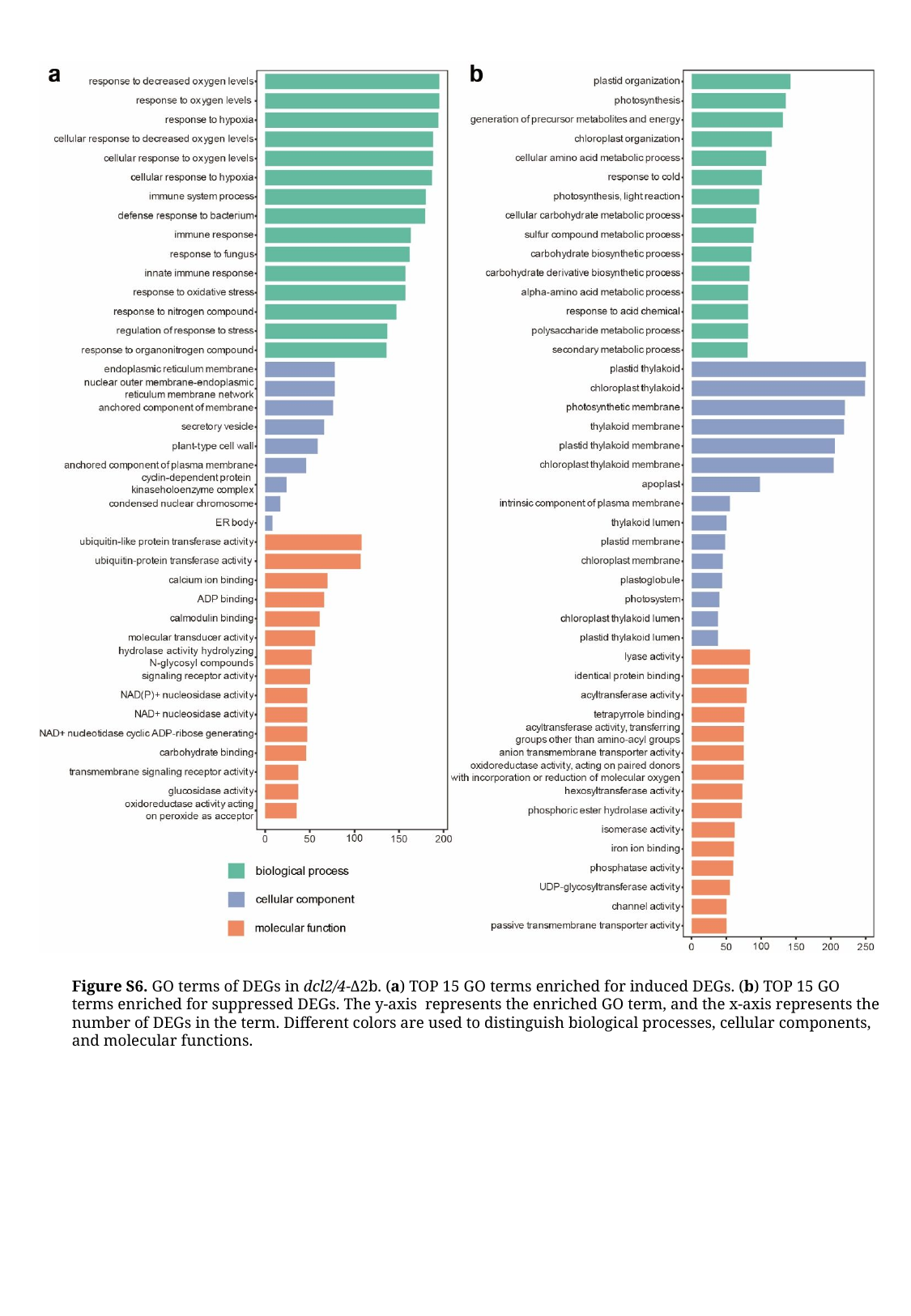

Figure S6. GO terms of DEGs in dcl2/4-Δ2b. (a) TOP 15 GO terms enriched for induced DEGs. (b) TOP 15 GO terms enriched for suppressed DEGs. The y-axis represents the enriched GO term, and the x-axis represents the number of DEGs in the term. Different colors are used to distinguish biological processes, cellular components, and molecular functions.

## Slide 7
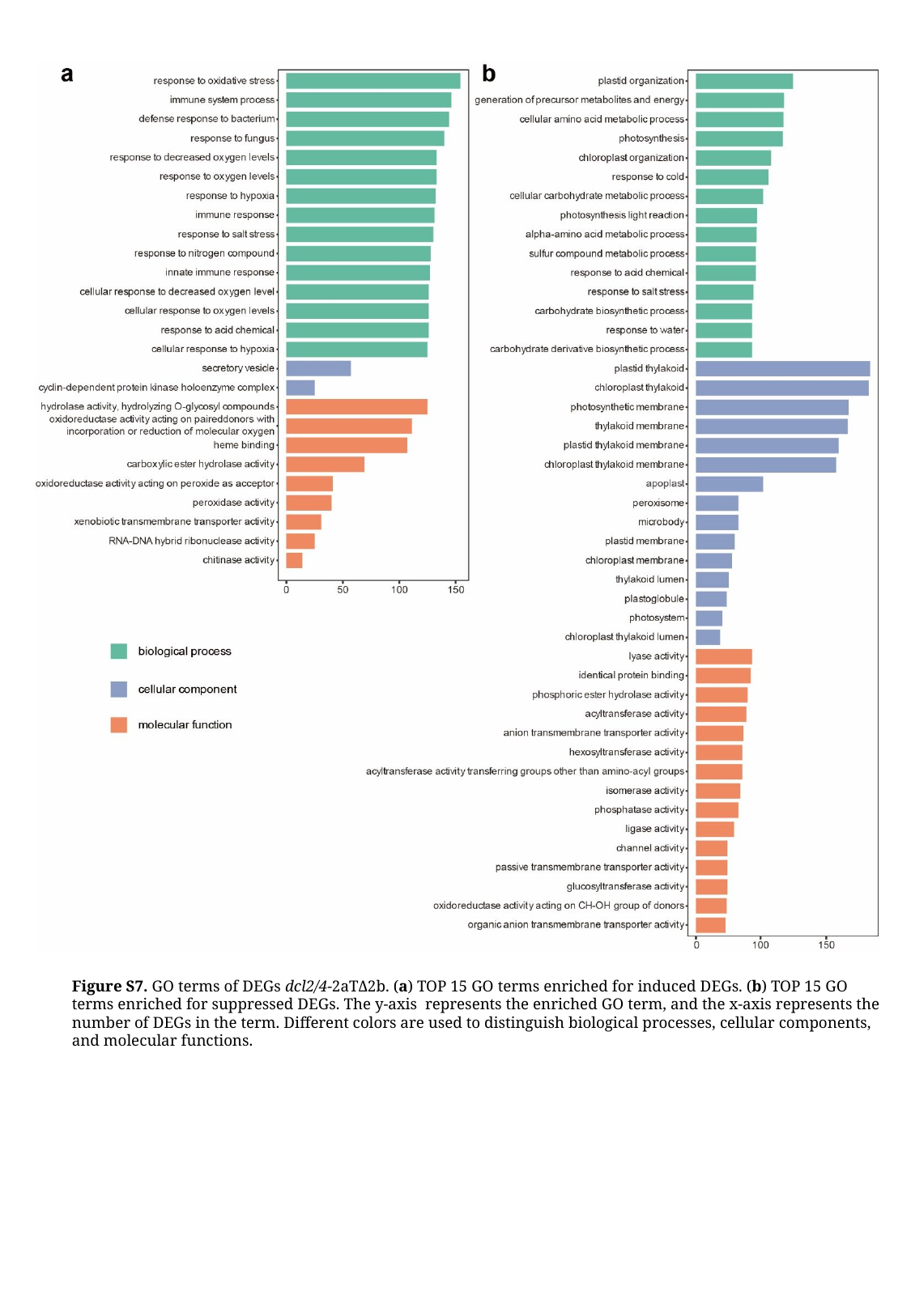

Figure S7. GO terms of DEGs dcl2/4-2aTΔ2b. (a) TOP 15 GO terms enriched for induced DEGs. (b) TOP 15 GO terms enriched for suppressed DEGs. The y-axis represents the enriched GO term, and the x-axis represents the number of DEGs in the term. Different colors are used to distinguish biological processes, cellular components, and molecular functions.
