# Supplementary material for: Comparative Transcriptome Analysis of CMV or 2b-Deficient CMV-Infected dcl2dcl4 Reveals the Effects of Viral Infection on Symptom Induction in Arabidopsis thaliana
Source: Viruses. 2022 Jul 21;14(7):1582. doi: 10.3390/v14071582 (PMC9320214; doi:10.3390/v14071582)
Supplement: Supplementary file 1 [file viruses-14-01582-s001.zip › Supplemetal Tables.pdf]

Table S1. Primers used for q-PCR.

Primer	Sequence（5'to3'）
AT1G31280-F	GGTGAACAACAGGATTTTCGAA
AT1G31280-R	CATAACTTCAGCTACACGAACG
AT1G33560-F	TGATGGAGATTGAGACGGTAAG
AT1G33560-R	TCATTCCAGAGATCCCAAACAA
AT3G52430-F	GAGTATCCCCGACAATAGCTAC
AT3G52430-R	TCTTTGACTAAAACGCCACTTG
AT1G73805-F	GCACTTATCGATGGTCATGTTC
AT1G73805-R	GTTCGCCCACTTCTAAAAAGTT
AT1G09350-F	ACCTTTTGCTGAACAGGATTTC
AT1G09350-R	ATATTTCCTTCTTGGCCTGTGA
AT5G03170-F	AAAGTTCAGCTTGTTCAGTTCC
AT5G03170-R	GTTACCGGAGCTAGTGATGTTA
AT4G38210-F	ACACTTGGAACTATCTCATGCC
AT4G38210-R	CACACTTGACCCTCCTATACTG
AT4G04330-F	AAACTCTGTAAGTGACGGTGAT
AT4G04330-R	GGCGCTTCATATTATCCCATTC
AT4G10250-F	CAGCAAGAGTAGACTGGAAAGA
AT4G10250-R	CTCTCCACTGACTCGTAAAACT
AT2G14610-F	TGGTCACTACACTCAAGTTGTT
AT2G14610-R	GCTTCTCGTTCACATAATTCCC
AT3G04720-F	ATAATCCGGCGCAGAATAATTG
AT3G04720-R	CAGTTACTGCAGCATTTGTTCT
AT1G75040-F	AGGATTTGAATTGACTCCAGGT
AT1G75040-R	CCATCGCCTACTAGAGTGAATT
AT3G57260-F	CGTTGTGGCTCTTTACAAACAA
AT3G57260-R	AGCTCTGAACGTTTTCTTGAAC
AT2G38010-F	TTAAGAACTCCTGGTCCAGAAC
AT2G38010-R	AGTGGCGATGTACTGAGAATAG

Table S2. Basic information of transcriptome sequencing data of Arabidopsis infected CMV or CMV mutants.

Sample	Clean Reads	GC Content(%)	Q20 (%)	Q30 (%)	Mapped Reads	Mapping Rate(%)
Col-Mock1	33995460	45.20	97.79	90.86	32679268	96.13
Col-Mock2	34165248	45.24	97.81	90.96	32823922	96.07
Col-Mock3	35502658	45.10	97.78	97.78	33961216	95.66
Col-CMV1	15307690	45.86	97.69	90.67	14685812	95.94
Col-CMV2	15301916	45.27	97.83	91.08	14605836	95.45
Col-CMV3	35337622	44.89	97.68	90.55	33745474	95.49
Col-Δ2b1	35732482	45.07	97.63	90.31	34556244	96.71
Col-Δ2b2	34038686	44.80	97.56	90.07	32802376	96.37
Col-Δ2b3	34107580	45.20	97.57	90.12	33027296	96.83
Col-2aTΔ2b1	34245920	45.86	97.69	90.67	31829350	92.94
Col-2aTΔ2b2	17136686	43.40	97.71	90.68	16094888	93.92
Col-2aTΔ2b3	35681694	43.31	97.85	91.04	33552040	94.03
dcl2/4-Mock1	34938414	45.70	97.38	89.52	33766308	96.65
dcl2/4-Mock2	34999866	45.88	97.42	89.70	33893420	96.84
dcl2/4-Mock3	34944582	45.64	97.47	89.88	33778854	96.66
dcl2/4- CMV 1	34877066	45.76	97.51	90.06	33313190	95.52
dcl2/4- CMV 2	34048228	45.11	97.79	90.64	32531898	95.55
dcl2/4- CMV 3	13921830	46.02	97.82	90.81	13354144	95.92
dcl2/4-Δ2b1	34123234	44.77	97.93	91.04	32821080	96.18
dcl2/4-Δ2b2	33948020	45.29	97.82	90.71	32814518	96.66
dcl2/4-Δ2b3	34056650	45.03	97.80	90.63	32570286	95.64
dcl2/4-2aTΔ2b1	34193226	42.50	98.02	91.34	31796116	92.99
dcl2/4-2aTΔ2b2	34292456	44.74	97.89	90.95	32384672	94.44
dcl2/4-2aTΔ2b3	35508300	42.98	97.89	90.90	32665494	91.99

Table S3. The log_2Foldchange of DEGs in all diseased plants.

Gene ID	Gene	Col-CMV	Col-Δ2b	Col-2aTΔ2b	*dcl2/4*-CMV	*dcl2/4*-Δ2b	*dcl2/4*-2aTΔ2b
AT2G30950	FTSH2	-1.04876	-0.60675	NA	-1.39758	-1.42179	-1.80262
AT1G12900	GAPA-2	-1.25864	-0.85696	-0.54100	-1.92817	-3.18391	-3.25448
AT1G07720	KCS3	-1.21285	-0.97109	NA	-1.52765	-2.31993	-2.13088
AT3G17470	ATCRSH	-1.32685	-0.63988	-0.93023	-1.23925	-1.97997	-2.47471
AT4G04640	ATPC1	-1.49321	-0.88481	-0.81764	-2.06362	-2.35429	-2.81177
AT3G52750	FTSZ2-2	-1.05207	NA	-0.72531	-1.77057	-1.90371	-2.43015
AT1G32080	AtLrgB	-1.29976	-0.59982	-0.38746	-1.92283	-2.80416	-2.70816
AT1G07640	OBP2	-1.44237	NA	NA	-1.63291	-1.11867	-1.45304
AT4G22890	PGR5-LIKE	-1.25374	-0.71086	-0.33962	-1.57654	-2.48210	-2.43964
AT5G54770	THI1	-1.44890	-0.87348	-0.72416	-1.49357	-2.00616	-1.74673
AT5G24420	PGL5	-1.15208	-0.50658	NA	-1.36096	-1.59649	-2.73878
AT5G67030	ABA1	-1.33716	-0.97256	-0.76666	-1.83942	-2.75672	-2.84931
AT3G19720	ARC5	-1.06646	-0.65804	-0.55477	-1.24006	-2.26606	-2.49429
AT1G16880	ACR11	-1.10228	-0.84804	-0.64912	-1.19544	-1.86118	-1.74655
AT5G48300	ADG1	-1.20211	-0.60178	-0.94243	-1.41065	-1.24277	-2.14099
AT4G20070	AAH	-1.08344	-0.57829	-0.51726	-1.59593	-2.70659	-2.52235
AT5G61670	AtOR	-1.24821	-0.93394	-0.64541	-1.20869	-1.66484	-1.70923
ATCG00120	ATPA	-1.03346	-0.41617	NA	-1.00661	-1.24554	-2.11498
ATCG00480	AthCF1beta	-1.42455	-0.55536	-0.92142	-1.18525	-1.67054	-2.84567
AT1G44446	ATCAO	-1.02378	-0.80732	-0.81811	-1.01996	-2.48119	-2.36303
AT3G63140	CSP41A	-1.43079	-0.82186	-0.35475	-1.66297	-2.30834	-2.20733
AT2G42540	AtCOR15A	-2.02238	NA	NA	-3.13781	-4.82777	-2.88397
AT2G31040	AtCGL160	-1.41613	-0.39767	-0.73105	-1.94273	-1.51812	-2.16621
AT3G01480	ATCYP38	-1.20314	-0.45078	-0.61049	-1.81795	-2.30881	-2.43874
AT1G03310	AtBE2	-1.26795	NA	NA	-1.95989	-2.85480	-1.76899
AT3G11670	DGD1	-1.14472	-0.74492	-0.87050	-1.32438	-1.90510	-1.86201
AT1G67440	emb1688	-1.44915	-0.82349	-0.86133	-1.52108	-1.75932	-1.80746
AT1G20020	ATLFNR2	-1.04490	NA	NA	-1.50278	-1.19606	-1.69052
AT5G23440	FTRA1	-1.03415	-0.92831	-0.58828	-1.30735	-1.21994	-1.31010
AT2G30390	ATFC-II	-1.23458	-0.62726	-0.80725	-1.81824	-2.50995	-2.49047
AT5G50950	FUM2	-2.12082	-0.63564	NA	-2.37357	-3.20742	-4.04507
AT1G09350	AtGolS3	-3.83435	NA	NA	-3.89427	-4.26067	-2.10429
AT5G62220	ATGT18	-1.20032	-0.63680	-0.86642	-2.23809	-3.49975	-2.85694
AT2G19860	ATHXK2	-1.35937	NA	-0.76298	-1.46012	-1.61906	-1.24551
AT3G17040	HCF107	-1.01524	-0.52371	-0.48287	-1.07159	-1.91689	-1.89647
AT1G18870	ATICS2	-2.15809	NA	NA	-1.93520	-3.26237	-1.72643
AT1G63970	ISPF	-1.01959	-0.42987	-0.47561	-1.16468	-1.08682	-1.30678
AT1G01790	ATKEA1	-1.24330	-0.67698	-0.73715	-1.59667	-2.29279	-2.29090
AT1G76570	AtLHCB7	-1.04371	-0.79122	NA	-1.38836	-2.44860	-2.66797
AT3G55760	LESV	-1.39029	0.00000	-0.60068	-1.61756	-1.12448	-1.10812
AT3G01510	LSF1	-1.27061	-0.66680	-0.82618	-1.54557	-1.57063	-1.60464

Gene ID	Gene	Col-CMV	Col-Δ2b	Col-2aTΔ2b	*dcl2/4*-CMV	*dcl2/4*-Δ2b	*dcl2/4*-2aTΔ2b
AT3G10940	LSF2	-1.43792	-0.57402	-0.59015	-1.96017	-2.97390	-2.36083
AT1G75690	LQY1	-1.22043	-0.73825	-0.26644	-1.36878	-2.18965	-2.05931
AT5G57030	LUT2	-1.23332	-0.60223	-0.37227	-1.37531	-2.14737	-2.08636
AT4G02530	MPH2	-1.38038	-0.75488	-0.65032	-1.42168	-1.63188	-1.94542
AT4G04040	MEE51	-1.48733	-0.52508	-0.70978	-1.37779	-1.33790	-1.82084
AT1G53240	mMDH1	-1.10707	-0.83871	-0.54307	-1.29735	-1.64643	-1.46537
AT1G31190	IMPL1	-1.01106	-0.76315	NA	-1.09728	-1.50925	-1.52705
AT5G10170	ATMIPS3	-1.44614	NA	-0.79191	-1.53583	-2.55402	-2.46920
AT1G32070	ATNSI	-1.05059	-0.53296	-0.63115	-1.20262	-1.15998	-1.34480
AT5G04900	NOL	-1.03262	-0.43222	-0.79471	-1.38799	-1.29300	-2.21014
AT5G51820	ATPGMP	-1.07773	NA	NA	-1.41774	-1.29045	-1.64058
AT4G24620	PGI	-1.00914	-0.58994	-0.57264	-1.18744	-1.53840	-1.79053
AT3G12780	PGK1	-1.18170	-0.82884	-0.43858	-1.39467	-1.70621	-1.80203
AT4G19100	PAM68	-1.01872	NA	-0.57171	-1.20303	-1.55338	-1.95818
AT3G16250	NDF4	-1.59442	-0.88650	-0.98740	-1.81428	-2.74441	-2.78688
AT1G18730	NDF6	-1.18690	NA	-0.82149	-1.81300	-2.21129	-2.92033
AT1G19150	LHCA2*1	-1.33323	-0.75068	-0.72271	-1.78648	-3.10848	-3.44945
ATCG00280	PSBC	-1.23660	NA	NA	-2.44630	-2.48612	-2.00217
AT2G20890	PSB29	-1.15741	-0.39141	-0.61774	-1.57128	-1.71879	-2.19199
AT1G06680	OE23	-1.14442	-0.78969	NA	-1.14361	-2.00531	-1.83156
AT4G05180	PSBQ	-1.24250	-0.68055	-0.54706	-1.35890	-1.84597	-2.31956
AT5G17230	PSY	-1.15109	-0.90603	-0.93453	-1.57380	-2.50134	-2.67229
AT1G66840	PMI2	-1.38539	-0.85803	-0.73038	-2.64067	-4.13796	-2.77461
AT3G52150	PSRP2	-1.18253	-0.31144	-0.45560	-1.69872	-1.38997	-1.97556
AT1G55480	ZKT	-1.50448	-0.91009	-0.82124	-1.78119	-2.35546	-2.80633
AT1G03630	POR	-1.39158	-0.77992	-0.82723	-1.95464	-2.35842	-2.78551
AT3G56650	PPD6	-1.00783	-0.48983	-0.46660	-1.05492	-1.12531	-1.49217
AT2G01918	PQL3	-1.18032	NA	NA	-1.40930	-2.93309	-1.96505
AT3G30720	QQS	-1.88113	NA	NA	-1.68412	-1.06196	-1.11014
AT5G66400	ATDI8	-2.27762	NA	NA	-1.51357	-2.66980	-2.82521
AT1G08540	ABC1	-1.03037	-0.49068	-0.48089	-1.45767	-1.76451	-2.19292
AT5G38410	RBCS3B	-1.03076	-0.80758	NA	-1.39400	-2.33631	-2.29586
AT5G40390	RS5	-1.58364	NA	NA	-2.16299	-2.39553	-1.95172
AT4G37930	SHM1	-1.34996	-0.93034	NA	-1.45300	-2.13841	-1.97538
AT1G64860	RPOD1	-1.31572	-0.97636	-0.84730	-1.49851	-2.54042	-2.55016
AT1G62750	ATSCO1	-1.20604	-0.95042	-0.82286	-1.37916	-2.25177	-2.33964
AT3G01180	AtSS2	-1.21451	-0.85151	-0.64847	-1.70786	-2.36700	-2.79689
AT1G44000	SGRL	-1.09824	-0.87139	-0.61330	-1.15711	-2.65538	-2.05908
AT1G68830	STN7	-1.24637	-0.89457	-0.90719	-1.26333	-1.55256	-1.91322
AT1G22710	ATSUC2	-1.30280	NA	-0.87167	-1.44032	-1.04949	-1.27605
AT1G03680	ATHM1	-1.19325	-0.69821	-0.50531	-1.02510	-1.05719	-1.47398
AT1G54780	AtTLP18.3	-1.62344	-0.70733	-0.93516	-1.89528	-2.16894	-2.58338

Gene ID	Gene	Col-CMV	Col-Δ2b	Col-2aTΔ2b	*dcl2/4*-CMV	*dcl2/4*-Δ2b	*dcl2/4*-2aTΔ2b
AT1G77490	TAPX	-1.34699	-0.51580	-0.79996	-2.23315	-2.47798	-3.46900
AT2G37720	TBL15	-1.63707	NA	NA	-3.27835	-4.85703	-2.68741
AT1G70230	AXY4	-1.42331	-0.59321	NA	-2.37823	-3.05013	-2.05810
AT5G05860	UGT76C2	-1.32012	-0.89368	NA	-1.26499	-2.60275	-2.14250
AT5G17790	OZ1	-1.02296	-0.69847	-0.64258	-1.26290	-1.62459	-1.59761
AT4G23160	CRK8	2.32010	NA	NA	4.70134	6.16321	5.36731
AT1G33560	ADR1	1.51739	NA	NA	2.08977	2.49932	2.26223
AT5G04720	ADR1-L2	1.20660	NA	NA	1.67173	1.92094	1.02334
AT1G31280	AGO2	1.94809	NA	-0.87971	3.23578	2.28215	1.47060
AT5G48380	BIR1	1.67029	NA	NA	2.84167	3.97184	2.83254
AT5G39670	CML46	1.65537	NA	NA	4.58765	5.60896	4.90176
AT4G23190	AT-RLK3	2.64969	0.00000	NA	3.18723	4.34096	1.62035
AT4G23210	CRK13	2.38546	NA	NA	4.32987	6.78806	5.20509
AT4G23230	CRK15	1.18755	NA	-1.85583	3.01389	4.03684	2.72085
AT4G23140	CRK6	2.00363	NA	-1.82166	4.19334	5.35433	4.04102
AT1G07000	ATEXO70B2	1.61871	NA	NA	3.35747	4.29040	4.19320
AT2G33340	MAC3B	1.18017	0.61277	0.60155	1.60850	1.44476	1.37918
AT5G46050	AtNPF5.2	2.03954	NA	NA	4.57864	6.71247	5.37613
AT3G52430	ATPAD4	1.44969	NA	NA	3.62058	4.90619	4.60006
AT2G32680	AtRLP23	1.43955	NA	-1.45041	4.01942	5.57942	4.95289
AT1G61380	LORE	1.06848	0.68970	NA	1.37083	2.71528	1.88122
AT1G73805	SARD1	2.44403	NA	NA	4.97588	5.72237	4.30534
AT5G14930	SAG101	1.41841	NA	NA	2.30638	3.73451	2.20859
AT5G25440	SZE1	1.58658	NA	NA	2.65150	3.08551	2.25368

Table S4. The log_2Foldchange of DEGs specifically detected in Col-CMV.

Gene ID	Gene	Col-CMV	Col-Δ2b	Col-2aTΔ2b	*dcl2/4*-CMV	*dcl2/4*-Δ2b	*dcl2/4*-2aTΔ2b
AT4G32460	BDX	1.632128	NA	0.801056	NA	NA	NA
AT3G55990	ESK1	1.459813	NA	NA	NA	NA	NA
AT4G38210	EXP20	3.406258	0	0	NA	NA	NA
AT4G38400	EXLA2	1.217828	NA	NA	0.891893	NA	NA
AT5G03170	FLA11	1.057445	NA	NA	NA	-1.31325	NA
AT1G17560	HLL	1.444426	NA	NA	NA	0.676283	NA
AT4G18780	CESA8	1.812452	NA	NA	NA	NA	NA
AT3G18660	GUX1	2.027156	NA	NA	NA	NA	NA
AT2G36570	PXC1	1.240795	0.834453	NA	NA	NA	NA
AT2G40320	TBL33	1.058063	NA	NA	NA	NA	NA
AT2G28315	UXT1	1.860752	NA	NA	NA	NA	NA
AT4G14130	XTH15	2.122686	NA	NA	NA	NA	NA
AT4G30290	XTH19	3.038013	NA	NA	NA	NA	-1.74706
AT2G06850	XTH4	1.380831	NA	NA	NA	NA	-1.13558
AT4G04330	RBCX1	-2.17407	1.228489	0.751557	-1.47059	0.717836	NA
AT3G63160	OEP6	-1.18245	NA	0.565459	-0.79089	-0.82909	-0.80548
AT4G39730	PLAT1	-1.43058	-0.8276	NA	-0.96338	-0.73827	NA
AT5G54640	HTA1	-1.27537	-0.76267	-0.53992	NA	NA	NA

Table S5. The log_2Foldchange of DEGs specifically detected in all virus-infected *dcl2/4*.

Gene ID	Gene	Col-CMV	Col-Δ2b	Col-2aTΔ2b	*dcl2/4*-CMV	*dcl2/4*-Δ2b	*dcl2/4*-2aTΔ2b
AT2G21280	ATSULA	0.60599	NA	NA	-1.05564	-1.26498	-1.36246
AT3G58830	PGPP1	0.67514	NA	NA	-1.21159	-1.49849	-1.60440
AT3G51895	AST12	NA	NA	NA	-1.10236	-1.88274	-1.09332
AT3G24590	PLSP1	0.85027	NA	0.33381	-1.24116	-1.42406	-1.47736
AT5G23120	HCF136	0.95808	0.44049	0.70353	-1.48939	-2.14683	-2.62188
AT4G09010	APX4	0.98644	NA	0.34682	-1.51266	-1.68008	-2.30445
AT3G08010	ATAB2	0.87238	0.29303	NA	-1.25156	-1.39727	-1.62154
AT3G20270	LBR-2	0.59267	NA	NA	-1.22588	-1.32030	-1.02218
AT5G55580	mTERF9	0.49025	NA	NA	-1.17393	-2.07937	-1.76588
AT5G55220	TIG1	NA	NA	-0.54060	-1.48510	-1.32948	-1.43937
AT5G21920	ATYLMG2	0.94606	0.39260	0.90199	-1.41813	-1.51593	-2.45753
AT1G10830	Z-ISO	0.64097	NA	NA	-1.06794	-1.01296	-1.50278
AT5G46290	KAS1	0.80291	0.43088	0.52533	-1.21307	-1.24720	-1.75900
AT2G16280	KCS9	0.48526	0.64162	0.76419	-1.22496	-2.24027	-2.10510
AT1G52340	ABA2	NA	NA	NA	-1.14240	-1.85265	-1.44181
AT5G65800	ACS5	0.00000	0.00000	0.00000	-2.55904	-4.01173	-4.63464
AT5G42480	ARC6	0.57443	NA	0.41324	-1.05258	-1.22216	-1.41788
AT4G25050	ACP4	0.79440	0.34448	NA	-1.25082	-1.81148	-1.71873
AT1G24490	ALB4	0.75570	NA	0.50500	-1.00166	-1.25890	-1.21065
AT4G35450	AFT	0.89284	0.63982	0.44253	-1.07500	-1.53118	-1.84916
AT5G65010	ASN2	NA	0.43445	0.55175	-1.02539	-1.69587	-1.18628
AT2G29940	ABCG31	NA	NA	NA	-2.48272	-2.03147	-1.84130
AT1G32500	ABCI7	0.78640	NA	NA	-1.26819	-1.22202	-1.36921
AT2G38120	AtAUX1	NA	NA	NA	-1.31771	-1.87043	-1.23636
AT4G33580	ATBCA5	0.75712	0.75866	0.82171	-1.13235	-2.14896	-2.24569
AT1G26560	BGLU40	0.75320	0.78122	0.83759	-1.57375	-2.97379	-2.86179
AT4G12030	BASS5	NA	NA	NA	-1.02524	-3.71998	-2.39437
AT3G29185	BFA1	0.45343	NA	NA	-1.00781	-1.30721	-1.64762
AT3G57180	BPG2	0.66460	NA	NA	-1.42553	-1.52415	-1.99805
AT5G23060	CaS	0.51045	NA	0.49334	-1.02022	-2.19333	-2.59028
AT1G06820	CCR2	0.54505	NA	0.67277	-1.15239	-1.21667	-2.00902
AT5G05270	AtCHIL	0.66318	0.90769	NA	-1.51741	-3.81225	-2.52115
AT1G25425	CLE43	0.95489	0.62128	NA	-1.14880	-2.30017	-1.57323
AT2G32950	ATCOP1	0.47062	0.42013	0.81324	-1.30963	-1.69331	-2.30000
AT3G56940	ACSF	0.82608	0.57568	0.32435	-1.20670	-2.20130	-2.25112
AT2G07050	CAS1	0.65985	NA	0.74824	-1.04912	-1.19359	-1.64877
AT2G31170	FIONA	NA	NA	NA	-1.06511	-1.27779	-1.22598
AT3G60620	CDS5	0.72456	NA	0.43202	-1.16336	-1.52817	-1.38749
AT4G00360	ATT1	NA	0.74680	0.85699	-1.29262	-2.29014	-2.54014
AT2G47940	DEG2	0.39600	-0.45073	0.68655	-1.11088	-1.08830	-2.00669

Gene ID	Gene	Col-CMV	Col-Δ2b	Col-2aTΔ2b	*dcl2/4*-CMV	*dcl2/4*-Δ2b	*dcl2/4*-2aTΔ2b
AT1G06080	ADS1	NA	NA	NA	-3.02635	-3.32595	-1.28777
AT1G80030	DJA7	0.77223	0.36981	NA	-1.09430	-1.74390	-1.45234
AT4G04350	EMB2369	0.65178	NA	0.39138	-1.20868	-1.37931	-1.71129
AT3G04340	emb2458	0.49005	NA	NA	-1.01418	-1.06393	-1.48638
AT5G18570	ATOBGC	NA	NA	NA	-1.37574	-1.31262	-1.66498
AT3G02660	EMB2768	NA	NA	NA	-1.08821	-1.19511	-1.63006
AT5G64580	EMB3144	0.68250	NA	NA	-1.75318	-1.49558	-2.00192
AT2G30200	EMB3147	0.97728	NA	0.46929	-1.19065	-1.04393	-1.79853
AT5G39980	EMB3140	NA	NA	NA	-1.32986	-1.45070	-1.05474
AT1G54040	ESP	0.50820	NA	NA	-1.00287	-1.65477	-2.18133
AT5G05740	ATEGY2	0.52768	NA	NA	-1.25779	-1.25850	-1.44289
AT2G45470	AGP8	NA	0.37134	NA	-1.96285	-3.70135	-2.72283
AT1G53520	AtFAP3	NA	-0.58228	-0.65115	-1.08133	-1.28531	-1.13783
AT5G66190	ATLFNR1	0.94837	0.40483	0.50172	-1.33357	-1.66489	-2.19604
AT3G02450	FtsHi3	0.74421	NA	NA	-1.28600	-1.65869	-1.61558
AT5G53170	FTSH11	0.56312	NA	0.80195	-1.06495	-1.18512	-2.18824
AT1G03160	FZL	0.91008	NA	0.44622	-1.12572	-1.28837	-1.57209
AT2G36830	GAMMA-TIP	0.60277	0.84079	0.45742	-1.31673	-3.42053	-2.56530
AT4G27600	NARA5	NA	NA	NA	-1.04116	-1.38589	-1.51041
AT5G13630	ABAR	0.87713	0.91440	0.67207	-1.02012	-2.09019	-2.49757
AT1G42540	ATGLR3.3	0.47151	NA	0.49378	-1.08649	-1.17425	-1.58525
AT1G42970	GAPB	0.98034	0.85445	0.82978	-1.42578	-2.30347	-2.68836
AT1G50900	GDC1	0.76600	NA	0.60447	-1.49150	-1.88056	-1.86530
AT3G28920	AtHB34	0.70725	0.47869	0.95117	-1.11338	-1.38016	-1.18156
AT3G11945	ATHST	0.87474	0.32135	0.78868	-1.37461	-1.42659	-2.01194
AT1G60990	IBA57.2	0.53792	NA	0.69983	-1.50902	-1.61365	-2.59966
AT4G17600	LIL3:1	NA	NA	NA	-1.10994	-1.63938	-1.59895
AT1G77590	LACS9	0.61588	0.40713	NA	-1.11927	-1.28357	-1.44799
AT3G46610	LPE1	0.61306	NA	NA	-1.03476	-1.55975	-1.43787
AT5G22830	ATMGT10	0.99415	NA	0.79349	-1.30956	-1.28607	-2.04130
AT3G16000	MFP1	0.78805	NA	NA	-1.19617	-1.62425	-1.19113
AT1G78610	MSL6	0.43175	NA	NA	-1.31581	-1.26343	-1.96454
AT5G11790	NDL2	0.94789	0.74394	0.77256	-1.02185	-2.02814	-1.78190
AT2G41680	NTRC	0.86821	0.41128	0.28462	-1.37523	-1.75740	-1.66937
AT2G38010	AtNCER2	0.62180	-0.54996	NA	-1.26153	-1.04076	-1.28374
AT5G65720	ATNFS1	0.41218	0.42714	0.75299	-1.04278	-1.19298	-1.33333
AT3G47450	ATNOA1	0.56200	NA	NA	-1.22990	-1.14139	-1.61424
AT1G44575	CP22	0.89200	0.81618	0.69051	-1.37762	-2.36861	-2.48162
AT1G62590	AtPPR-AC	NA	NA	NA	-1.40011	-1.98057	-2.77214
AT3G23430	ATPHO1	NA	NA	NA	-1.03074	-2.35967	-1.61599
AT2G39470	PnsL1	0.99343	0.57695	0.38307	-1.16182	-2.15708	-1.93570

Gene ID	Gene	Col-CMV	Col-Δ2b	Col-2aTΔ2b	*dcl2/4*-CMV	*dcl2/4*-Δ2b	*dcl2/4*-2aTΔ2b
AT5G58140	AtPHOT2	0.76245	NA	0.87168	-1.28799	-2.26952	-2.84889
AT1G23080	ATPIN7	0.43053	NA	0.44583	-1.52126	-2.66745	-1.65916
AT4G23400	PIP1;5	0.77642	0.83168	0.70343	-1.14196	-1.91003	-1.29516
AT5G38150	PMI15	0.86717	NA	NA	-1.67787	-1.68019	-1.16841
AT1G64510	PRPS6	0.89074	0.38598	0.51446	-1.03155	-1.15576	-1.73189
AT5G54180	PTAC15	0.64864	NA	NA	-1.25582	-1.51809	-2.22753
AT4G13670	PTAC5	0.67025	NA	0.41422	-1.04764	-1.45371	-1.63871
AT3G55330	PPL1	0.78259	0.35493	NA	-1.04331	-1.75911	-1.72549
AT3G21110	ATPURC	NA	NA	NA	-1.06318	-1.53362	-1.75767
AT4G04340	osca1	0.81504	NA	NA	-1.06056	-1.20623	-1.41915
AT4G02260	AT-RSH1	0.74857	NA	NA	-1.04727	-1.40566	-1.44107
ATCG00780	RPL14	0.62135	NA	-0.30325	-1.21774	-1.28826	-1.28349
AT3G63190	AtcpRRF	0.75936	NA	NA	-1.17667	-1.19625	-1.07570
AT3G62030	CYP20-3	0.86940	0.35853	NA	-1.19426	-1.23406	-1.30787
AT3G25570	SAMDC3	0.99039	NA	NA	-1.33779	-1.87794	-1.04925
AT2G35500	SKL2	0.64186	NA	NA	-1.14968	-1.03038	-1.55096
AT1G11720	ATSS3	0.64613	NA	NA	-1.20886	-1.57465	-1.93064
AT2G46340	SPA1	0.94975	0.42718	0.76708	-1.80864	-2.38923	-1.66865
AT2G43710	AtSSI2	0.96031	0.81477	0.68236	-1.26602	-2.14193	-2.01690
AT5G13650	SVR3	0.86169	0.61677	0.70137	-1.65397	-2.37822	-2.70456
AT4G16390	SVR7	0.86171	NA	0.53370	-1.35325	-1.22085	-1.22853
AT4G18390	TCP2	NA	NA	NA	-1.09427	-1.73268	-1.12367
AT5G53490	TL17	0.91271	NA	NA	-1.19422	-1.47353	-1.73913
AT5G16620	ATTIC40	NA	NA	-0.41187	-1.01148	-1.00797	-1.30961
AT2G30460	UXT2	0.75535	NA	0.71616	-1.36815	-1.63097	-1.76911
AT5G11230	UAFT2	NA	NA	NA	-1.21558	-1.41544	-1.16734
AT4G12490	AZI3	0.00000	0.00000	0.00000	6.19668	10.15793	10.19413
AT1G73260	ATKTI1	0.00000	0.00000	0.00000	4.21099	8.13837	11.11922
AT2G14610	AtCAPE9	NA	NA	3.64813	4.88181	9.13765	9.18787
AT1G66700	PXMT1	NA	NA	NA	5.48484	7.62354	9.74821
AT5G64905	PROPEP3	NA	0.00000	0.00000	6.64256	9.16496	6.89509
AT4G12480	EARLI1	0.00000	0.00000	0.00000	3.87684	8.46059	7.93555
AT2G43510	ATTI1	NA	NA	NA	4.86098	7.03814	7.67610
AT1G79680	ATWAKL10	NA	0.00000	NA	4.21513	7.68951	6.24356
AT3G57260	AtBG2	NA	NA	NA	4.77443	6.85092	6.49609
AT2G43570	CHI	NA	NA	NA	4.42596	6.52516	6.97102
AT4G10250	ATHSP22.0	0.00000	0.00000	0.00000	5.88810	6.46311	5.06256
AT2G29110	ATGLR2.8	NA	0.00000	NA	4.85827	7.20545	5.10202
AT2G35980	ATNHL10	NA	0.00000	NA	3.77181	6.87019	5.78828
AT1G32960	ATSBT3.3	NA	NA	NA	4.50433	6.33515	5.50720
AT4G21680	AtNPF7.2	0.00000	0.00000	NA	4.59660	6.48820	5.14498
AT5G11210	ATGLR2.5	NA	NA	NA	3.06175	6.57890	6.56110

Gene ID	Gene	Col-CMV	Col-Δ2b	Col-2aTΔ2b	*dcl2/4*-CMV	*dcl2/4*-Δ2b	*dcl2/4*-2aTΔ2b
AT4G11470	CRK31	NA	NA	NA	4.28819	6.37789	5.17253
AT1G34180	anac016	NA	0.00000	NA	4.34604	6.26161	5.21563
AT3G46230	ATHSP17.4	NA	0.00000	0.00000	6.47538	5.04776	4.14287
AT3G44300	AtNIT2	NA	NA	NA	3.32235	6.11269	6.06624
AT4G27460	CBSX5	NA	0.00000	NA	4.83269	5.29418	5.05758
AT4G23310	CRK23	NA	NA	2.93813	4.33907	6.41884	4.10503
AT1G68765	IDA	0.00000	0.00000	0.00000	4.16282	6.10906	4.03205
AT4G13890	EDA36	0.00000	0.00000	0.00000	2.76216	5.44734	5.88654
AT4G23150	CRK7	NA	NA	2.76986	3.90315	5.76861	4.42110
AT1G26380	AtBBE3	NA	NA	3.78407	2.93682	5.87600	4.37102
AT5G10380	ATRING1	NA	NA	0.57411	3.16746	4.76171	5.11837
AT2G26560	PLA	NA	NA	NA	2.76983	6.36949	3.82957
AT1G75040	PR-5	NA	NA	1.12637	2.70642	4.77103	5.44977
AT3G09940	AtMDAR3	NA	NA	0.00000	2.75723	5.38246	4.60085
AT1G09080	BIP3	NA	NA	1.88669	3.40125	4.40708	4.84479
AT4G11170	RMG1	NA	NA	NA	2.33318	5.48496	4.70810
AT4G10500	DLO1	2.28106	NA	NA	2.64313	4.52412	5.21030
AT5G48650	G3BP-like	NA	0.00000	NA	4.30746	4.14830	3.80342
AT5G64810	ATWRKY51	NA	NA	2.53294	3.43886	4.53434	4.24618
AT4G02520	ATGSTF2	NA	1.27794	1.54688	3.45429	4.14387	4.53707
AT4G21840	ATMSRB8	2.40064	2.81191	3.75586	2.33354	4.81725	4.76287
AT5G15120	PCO1	NA	NA	NA	2.86096	6.16492	2.83218
AT5G26170	ATWRKY50	-0.68564	NA	NA	3.05418	4.45822	4.22429
AT1G03850	ATGRXS13	NA	NA	1.83424	2.67586	5.00151	4.04925
AT2G47130	AtSDR3	NA	0.90420	1.43708	3.18045	4.11002	4.12319
AT5G55460	AtLTP4.5	NA	NA	1.64023	2.95695	4.33166	4.06777
AT2G35000	ATL2G	NA	0.00000	NA	2.91614	4.31834	4.10813
AT5G39610	ANAC092	0.00000	0.00000	0.00000	2.58767	4.44464	4.09389
AT5G01900	ATWRKY62	NA	NA	NA	2.57532	4.62707	3.90998
AT3G59700	ATHLECRK	NA	NA	NA	2.93610	4.72406	3.37562
AT3G04720	AtPR4	NA	0.91583	1.90269	2.18542	3.76049	4.24530
AT4G33050	AtIQM1	NA	NA	NA	2.98640	4.17679	2.84096
AT4G26270	PFK3	NA	NA	NA	2.10330	3.88477	3.39752
AT3G11840	PUB24	NA	NA	NA	2.35025	4.10999	2.48443
AT4G21390	B120	NA	NA	NA	2.97965	4.17904	1.71136
AT1G06160	ERF59	NA	NA	NA	3.28225	2.55718	2.96674
AT4G34390	XLG2	NA	NA	NA	2.64302	3.78729	2.26242
AT1G08450	AtCRT3	-0.99135	NA	NA	2.34064	3.26907	3.06249
AT2G19990	PR-1-LIKE	NA	0.00000	NA	3.23959	2.40547	2.70272
AT5G22570	ATWRKY38	NA	NA	NA	1.72395	3.20000	3.29246
AT3G50470	HR3	-0.79690	NA	1.10074	2.58120	3.17874	2.32314
AT2G48150	ATGPX4	0.00000	0.00000	NA	2.54523	3.01972	2.47883

Gene ID	Gene	Col-CMV	Col-Δ2b	Col-2aTΔ2b	*dcl2/4*-CMV	*dcl2/4*-Δ2b	*dcl2/4*-2aTΔ2b
AT5G38280	PR5K	NA	NA	NA	2.21096	3.36248	2.40664
AT3G07520	ATGLR1.4	-0.69378	NA	NA	2.15715	3.11533	2.64371
AT1G02930	ATGST1	NA	2.14482	2.61516	2.08011	3.61524	2.15545
AT1G51800	IOS1	NA	NA	1.44195	1.76504	3.88558	2.19472
AT2G25000	ATWRKY60	NA	NA	1.26271	1.61444	3.02953	3.18640
AT3G51430	SSL5	NA	NA	NA	1.61083	3.00161	3.04524
AT3G16030	CES101	NA	NA	NA	2.39182	3.43014	1.70776
AT4G39030	EDS5	NA	NA	NA	1.57057	3.50910	2.38470
AT1G62300	ATWRKY6	NA	NA	NA	1.47812	3.55834	2.28345
AT3G54420	ATCHITIV	NA	0.00000	NA	2.23527	3.07039	1.87836
AT3G45860	CRK4	NA	NA	2.39117	1.98025	3.04412	1.98805
AT1G70690	HWI1	NA	NA	NA	1.38929	2.31288	3.30669
AT3G12500	ATHCHIB	NA	NA	NA	1.84109	2.18359	2.89058
AT5G35580	PBL13	NA	0.00000	NA	1.97439	3.00941	1.88873
AT1G24150	ATFH4	NA	NA	NA	1.94436	3.15763	1.73134
AT3G25020	AtRLP42	NA	NA	1.28959	1.43078	2.54265	2.84150
AT4G33300	ADR1-L1	-0.95706	NA	NA	1.97530	2.67538	2.09459
AT1G77120	ADH	NA	NA	NA	1.58389	3.56208	1.54270
AT5G60280	LecRK-I.8	-0.77698	NA	NA	1.66683	2.69081	2.12809
AT1G74710	ATICS1	NA	0.67872	1.04857	1.25502	3.04154	2.16926
AT2G31870	atPARG1	-0.77769	-0.88800	NA	1.92914	2.62249	1.87891
AT3G45060	ATNRT2.6	NA	NA	NA	1.76446	2.56857	1.93493
AT4G30370	IDF1	NA	NA	NA	1.52615	1.91353	2.77055
AT3G13437	AtEWR1	NA	NA	1.46575	1.73343	2.96613	1.47758
AT3G14990	AtDJ1A	-0.99973	NA	NA	1.47868	2.56107	2.08001
AT1G63460	ATGPX8	-0.80650	NA	NA	1.82688	2.09707	2.15711
AT1G07260	UGT71C3	NA	NA	NA	1.50394	1.75944	2.64793
AT5G20250	DIN10	NA	NA	NA	1.84889	2.51312	1.54512
AT3G07525	ATATG10	NA	NA	NA	1.22515	1.98609	2.62613
AT3G59660	BAGP1	NA	NA	NA	1.30777	2.51778	1.98800
AT1G17600	SOC3	NA	NA	0.79065	1.76793	2.29470	1.70773
AT3G53230	AtCDC48B	NA	0.76303	NA	1.52621	1.98517	2.25522
AT3G48090	ATEDS1	NA	NA	NA	1.84994	2.16398	1.73318
AT1G21250	AtWAK1	NA	0.66828	1.68633	1.51317	1.91823	2.21596
AT3G20250	APUM5	NA	NA	NA	1.34951	2.55312	1.71931
AT4G03960	AtPFA-DSP4	NA	NA	NA	1.46182	1.93360	2.15744
AT3G45590	ATSEN1	NA	NA	NA	1.39062	1.93826	2.18657
AT5G24530	AtDMR6	-0.65303	NA	1.37507	1.63013	2.07627	1.74156
AT4G14430	ATECI2	-0.36548	NA	-0.56686	1.31937	1.72528	2.39008
AT3G55450	PBL1	-0.66365	-0.98510	NA	1.26787	2.26598	1.77271
AT4G23260	CRK18	0.55447	1.27219	1.21631	1.21510	2.67646	1.31557

Gene ID	Gene	Col-CMV	Col-Δ2b	Col-2aTΔ2b	*dcl2/4*-CMV	*dcl2/4*-Δ2b	*dcl2/4*-2aTΔ2b
AT4G21980	APG8A	NA	NA	NA	1.12282	1.95575	2.10795
AT3G25882	NIMIN-2	NA	NA	NA	1.28842	1.84293	2.04130
AT1G53320	AtTLP7	-0.67431	-0.63760	-0.89198	1.44554	1.74842	1.78624
AT3G12490	ATCYS6	-0.69963	-0.64231	-0.83653	1.40849	1.74314	1.82361
AT5G07470	ATMSRA3	-0.36689	NA	NA	1.37025	1.53703	2.00064
AT3G63080	ATGPX5	NA	0.47707	NA	1.20444	1.64963	2.03775
AT5G27100	ATGLR2.1	NA	NA	NA	1.52459	1.35260	2.01343
AT5G01560	LecRK-VI.4	NA	NA	0.55149	1.47541	1.33489	2.01562
AT2G43850	ILK1	NA	NA	NA	1.11154	2.04573	1.62960
AT2G43350	ATGPX3	-0.65022	-0.52952	NA	1.31151	1.65214	1.78308
AT2G19810	AtOZF1	-0.75251	-0.73779	NA	1.62744	1.39345	1.69383
AT1G65980	TPX1	-0.51934	NA	-0.48052	1.41936	1.38707	1.88822
AT5G45250	RPS4	-0.51313	NA	NA	1.27194	1.67947	1.71125
AT4G22305	SOBER1	NA	0.63969	1.25951	1.39176	1.50166	1.75226
AT5G43170	AZF3	NA	NA	NA	1.57396	1.32083	1.73809
AT2G25110	AtSDF2	-0.50299	NA	NA	1.03041	1.77167	1.80521
AT5G37055	ATSWC6	-0.86416	NA	-0.74127	1.48909	1.26024	1.83304
AT4G04920	AtSFR6	-0.92241	NA	-0.51070	1.12654	1.72298	1.63719
AT3G63420	AGG1	NA	0.44099	NA	1.38121	1.55668	1.53949
AT2G01340	At17.1	NA	NA	NA	1.20902	1.40753	1.85250
AT4G23240	CRK16	NA	NA	NA	1.48976	1.72115	1.11065
AT3G55460	At-SCL30	-0.69870	NA	-0.49296	1.47558	1.32238	1.52159
AT5G48810	ATB5-B	-0.57471	NA	-0.55947	1.31650	1.22399	1.71824
AT1G64280	ATNPR1	NA	NA	NA	1.24864	1.63524	1.12819
AT4G03510	ATRMA1	NA	NA	NA	1.16303	1.22224	1.56059
AT1G52740	H2A.Z	-0.85375	-0.47628	-0.99045	1.14798	1.26534	1.27041
AT1G09415	NIMIN-3	-0.45902	-0.47260	-0.97724	1.43799	1.07643	1.04635
AT5G63030	GRXC1	NA	0.51488	NA	1.15167	1.15665	1.17257
